# Knockdown of the long noncoding RNA PURPL induces apoptosis and sensitizes liver cancer cells to doxorubicin

**DOI:** 10.1038/s41598-022-23802-9

**Published:** 2022-11-14

**Authors:** Tsinat Berhane, Anja Holm, Kasper Thystrup Karstensen, Andreas Petri, Mirolyuba Simeonova Ilieva, Henrik Krarup, Mogens Vyberg, Marianne Bengtson Løvendorf, Sakari Kauppinen

**Affiliations:** 1 Center for RNA Medicine, Department of Clinical Medicine, Aalborg University, Copenhagen, Denmark; 2 Present Address: Department of Health Technology, Technical University of Denmark, Kgs. Lyngby, Denmark; 3 Present Address: Department of Bacteria, Parasites and Fungi, Statens Serum Institute, Copenhagen, Denmark; 4 Present Address: Department of Bioinformatics, Lundbeck, Valby, Denmark; 5 Department of Molecular Diagnostics and Department of Medical Gastroenterology, Aalborg University Hospital, and Department of Clinical Medicine, Aalborg University, Aalborg, Denmark; 6 Department of Pathology, Aalborg University Hospital, and Department of Clinical Medicine, Aalborg University, Aalborg, Denmark; 7 Present Address: Department of Dermatology and Allergy, Herlev and Gentofte Hospital, University of Copenhagen, Hellerup, Denmark

**Keywords:** Cancer, Non-coding RNAs, Transcriptomics

## Abstract

Hepatocellular carcinoma (HCC) is the most common type of primary liver cancer with increasing incidence in western countries. Most HCC patients have advanced cancer at the time of diagnosis due to the asymptomatic nature of early-stage HCC and do not qualify for potentially curative surgical treatment, thus, highlighting the need for new therapeutic strategies. Long noncoding RNAs (lncRNAs) comprise a large and heterogeneous group of non-protein coding transcripts that play important regulatory roles in numerous biological processes in cancer. In this study, we performed RNA sequencing of liver biopsies from ten HCC, ten hepatitis C virus-associated HCC, and four normal livers to identify dysregulated lncRNAs in HCC. We show that the lncRNA p53-upregulated-regulator-of-p53-levels (PURPL) is upregulated in HCC biopsies and that its expression is p53-dependent in liver cancer cell lines. In addition, antisense oligonucleotide-mediated knockdown of PURPL inhibited cell proliferation, induced apoptosis, and sensitized HepG2 human HCC cells to treatment with the chemotherapeutic agent doxorubicin. In summary, our findings suggest that PURPL could serve as a new therapeutic target for reversing doxorubicin resistance in HCC.

## Introduction

Liver cancer is the third leading cause of cancer-related deaths worldwide with approximately 905,677 new cases and 830,180 deaths reported in 2020^1^, and with a 5-year survival rate of less than 20% depending on stage of cancer^2^. HCC comprises 75–85% of all liver cancers and is thereby the most common type of liver cancer with increasing incidence primarily in western countries^3,4^. Major risk factors include cirrhosis, and hepatitis B and C virus infection. Other risk factors associated with HCC tumorigenesis include diabetes, smoking, obesity, alcohol abuse, and non-alcoholic steatohepatitis (NASH)^3,5^. HCC is highly resistant to therapy and therefore challenging to treat^6,7^. Liver transplantation, hepatectomy, and local ablation remain optimal treatment options for early-stage HCC patients, who show no signs of inadequate liver function, portal hypertension, or metastasis. However, most HCC patients have advanced cancer at the time of diagnosis and do not qualify for these treatment options^5,8,9^.

Recent data imply that lncRNAs play important regulatory roles in normal physiology and human disease, including cancer^10^. LncRNAs comprise a large, heterogeneous group of non-protein coding transcripts longer that 200 nucleotides in length. In addition, they are less conserved than mRNAs and often expressed at lower levels than protein-coding genes^11–14^. The metastasis-associated lung adenocarcinoma transcript 1 (MALAT1) was among the first human lncRNAs to be identified in cancer due to its high expression in metastatic lung cancer cells^15^. In addition, ASO-mediated knockdown of MALAT1 reduced cell proliferation, survival, migration, invasion as well as metastasis in immunocompromised mice^16^, highlighting its potential as a therapeutic target in cancer. Thus, identification and characterization of the pathophysiological functions of lncRNAs may provide new therapeutic strategies for cancer^16,17^.

Several lncRNAs have been implicated in the pathogenesis of HCC. The expression of the lncRNA antisense non-coding RNA in the INK4 locus (ANRIL) is significantly upregulated in HCC compared to adjacent normal liver tissue samples. Knockdown of ANRIL in vivo suppressed cell proliferation, metastasis, and invasion by regulating miR-122-5p expression^18^. The lncRNA taurine up-regulated 1 (TUG1) is highly expressed in HCC, and its knockdown suppresses cell migration, invasion, metastasis, and glycolysis^19^. The HOX transcript antisense intergenic RNA (HOTAIR) is upregulated in HCC, and knockdown of HOTAIR suppressed cell proliferation and invasion in vitro as well as the growth of liver cancer cells in vivo. Moreover, high HOTAIR expression has been linked to shorter recurrence-free survival compared to patients with low HOTAIR levels^20,21^.

In the present study, we carried out RNA sequencing (RNAseq) of biopsies from liver cancer patients to identify differentially expressed lncRNAs in HCC. We show that the lncRNA PURPL is significantly upregulated in both virus-unrelated HCC and in HCV-associated HCC compared to normal liver tissue. Furthermore, ASO-mediated knockdown of PURPL inhibited cell proliferation and induced apoptosis. Interestingly, a decreased level of PURPL mediated by ASOs enhances the response to the chemotherapeutic agent doxorubicin (DOX) in cultured liver cancer cells, suggesting that PURPL may have therapeutic potential in the treatment of chemoresistant HCC.

## Results

### PURPL is upregulated in hepatocellular carcinoma

**Figure 1:**
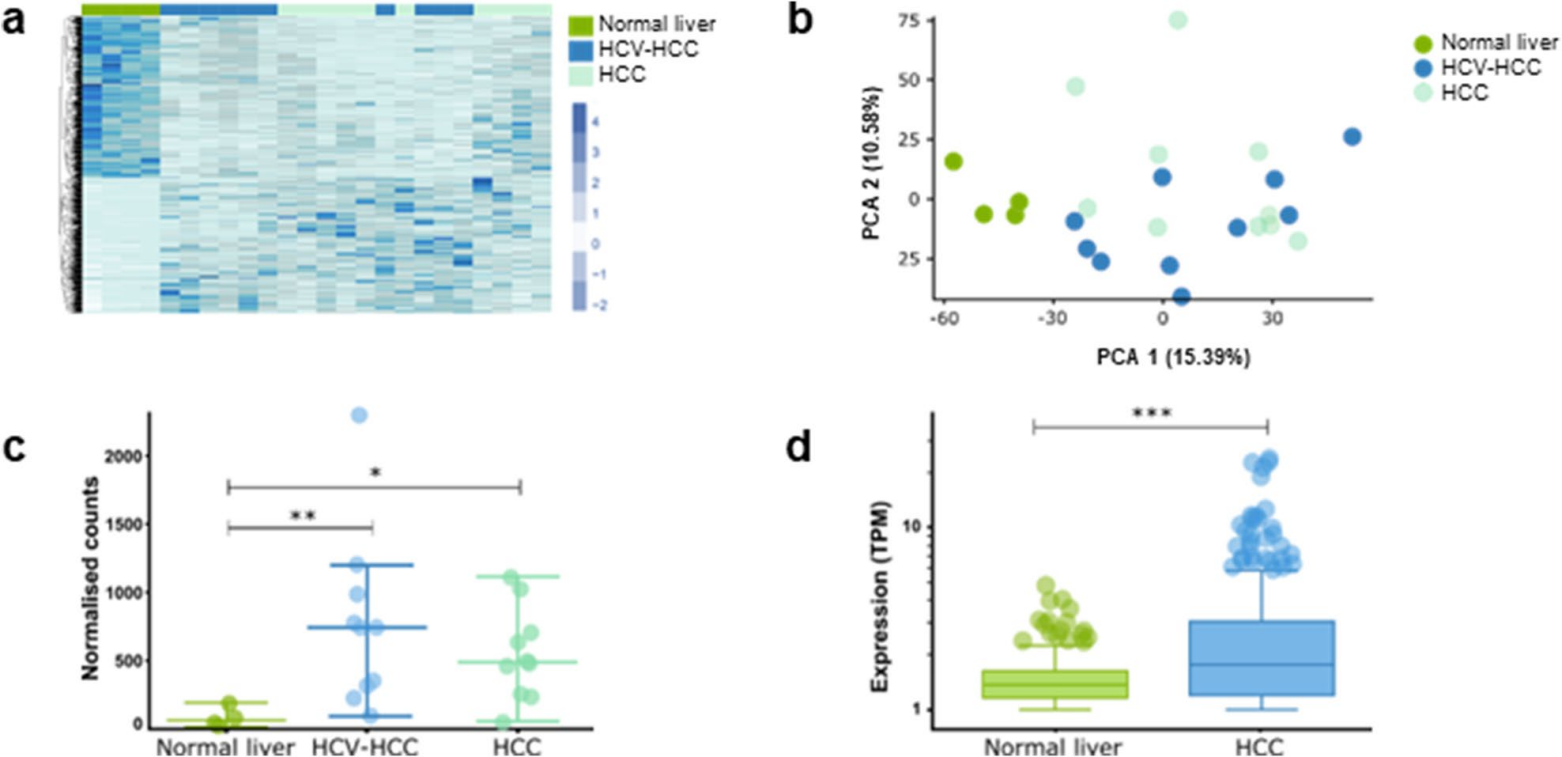
Identification and characterization of the lncRNA PURPL in HCC. (**a**) Heat map of unsupervised hierarchical clustering of normalized gene counts for all significant differentially expressed genes across the sequenced liver biopsies (FC > 1.5). Genes are sorted from highest to lowest adjusted p-value (*P* < 0.05). Normal liver (*n* = 4; green), HCV-HCC (*n* = 10; blue), HCC (*n* = 10; light blue). (**b**) The first and second principal component of the gene expression matrices of each liver biopsy group are shown. Normal liver (*n* = 4; green), HCV-HCC (*n* = 10; blue), HCC (*n* = 10; light blue). (**c**) T-test between PURPL expression in normal liver tissue (*n* = 4) and each of the HCC groups showed a significantly increased expression of PURPL in the HCC (*n* = 10; *P* = 4.29E-2) and the HCC-HCV groups (*n* = 10; *P* = 2.84E-4). (**d**) TCGA and GTex data of PURPL expression levels in liver cancer tissue (*n* = 369) and normal liver tissue (*n* = 160). T-test between groups showed a significantly increased PURPL expression in liver cancer. The data were extracted from the open-access portal MiPanda.

To identify dysregulated lncRNAs in HCC, we performed total RNAseq of liver biopsies from ten patients with virus-unrelated HCC (termed HCC), ten patients with HCV-associated HCC (HCV-HCC), and four subjects with normal liver. Unsupervised hierarchical clustering and principal component analysis (PCA) of the expression profiles differentiated the normal liver biopsies from the two HCC diagnostic groups (Fig. 1a,b). By performing pairwise comparison of the expression profiles between the normal liver biopsies to those of the HCC and HCV-HCC biopsies, we identified 240 and 258 (*P* < 0.05, FC > 1.5; Supplementary Table S1 + Supplementary Fig. S1a + b) differentially expressed genes, respectively. Among the list of differentially expressed genes, we identified 13 lncRNAs in the data set including AL365181.3, HELLP associated long non-coding RNA (*HELLPAR*), and the lncRNA p53-upregulated-regulator-of-p53-levels (*PURPL*; Table 1). The lncRNA AL365181.3 was the most upregulated lncRNA in HCC relative to normal liver tissue, whereas *HELLPAR* was the most upregulated lncRNA in HCV-HCC relative to normal liver samples (Table 1). However, the lncRNA *PURPL* was the most upregulated lncRNA in both HCC groups relative to normal liver tissue and was therefore chosen for further studies (Fig. 1c; Table 1). *PURPL* has previously been shown to be associated with the tumor suppressor p53 and to promote tumorigenesis in colorectal cancer (CRC)^22,23^ and HCC^24^. We confirmed increased expression of *PURPL* in HCC using data from The Cancer Genome Atlas (TCGA) and Genetype-Tissue Expression (GTEx) database (*P* = 4.10E-8, TCGA-Liver Hepatocellular Carcinoma (LIHC): *n* = 369, Normal liver tissue: *n* = 160) (Fig. 1d). Since PURPL is transcriptionally regulated by p53^23^ and its expression correlates with p53 expression in our data set (r = 0.4065, *P* = 0.0487; Supplementary Fig. S1c), we also assessed expression of the p53-regulated genes; p53 binding protein 1 and 2 (TP53BP1, TP53BP1), p21 (CDKN1A), mouse double minute 2 homolog (MDM2), and BCL-2 associated X (BAX) in the same liver samples. Interestingly, expression of the selected p53-regulated genes were upregulated in HCC liver samples compared to control livers. However, this did not reach statical significance, except for TP53BP, which was significantly upregulated in the HCC liver tissue (*P* = 1.59E-02) and HCC-HCV liver tissue (*P* = 0.006) compared to normal liver samples (Supplementary Fig. S1d-i).

**Table 1:** Differentially expressed lncRNAs in HCC biopsies relative to normal liver tissue.

Gene ID			HCV-HCC		HCC	
Ensembl gene ID	Gene name	Biotype	Log2 fold change	Adjusted *P* value	Log2 fold change	Adjusted *P* value
ENSG00000272405	AL365181.3	Antisense			6.3816	1.031e-02
ENSG00000281344	HELLPAR	Macro lncRNA	3.298	1.190e-02		
ENSG00000250337	PURPL	lincRNA	3.064	2.841e-03	2.426	4.292e-02
ENSG00000249859	PVT1	lincRNA	2.599	8.724e-03	2.237	4.837e-02
ENSG00000228526	MIR34AHG	lincRNA	2.391	3.614e-05	2.262	2.444e-04
ENSG00000172965	MIR4435-2GH	lincRNA	2.220	1.743e-03	2.089	6.562e-03
ENSG00000222041	CYTOR	lincRNA	1.814	7.270e-03	1.736	1.782e-02
ENSG00000251562	MALAT1	lincRNA	1.616	1.244e-04		
ENSG00000259820	AC083843.3	lincRNA	1.590	1.061e-02		
ENSG00000284959	AC007262.2	Antisense			− 1.547	1.197e-02
ENSG00000265735	RN7SL5P	misc_RNA	− 2.167	3.880e-02		
ENSG00000263740	RN7SL4P	misc_RNA	− 2.373	3.245e-02		
ENSG00000274012	RN7SL2	misc_RNA	− 2.557	9.554e-05		

Additionally, we quantified *PURPL* in two p53 wild-type cancer cell lines, HepG2 (human HCC cell line) and Sk-hep-1 (human hepatic adenocarcinoma cell line), and the immortalized human liver cell line Thle-3. *PURPL* was upregulated by 19.5- and 43.6-fold, respectively, in HepG2 and Sk-hep-1 cells, compared to Thle-3 cells (Supplementary Fig. S2a). Furthermore, we showed that PURPL localizes primarily into the nucleus (77.1%) (Supplementary Fig. S2b), which is consistent with previous reports in CRC cell lines^22,23^.

### Knockdown of* PURPL* induces apoptosis and decreases proliferation of cultured liver cancer cells

**Figure 2:**
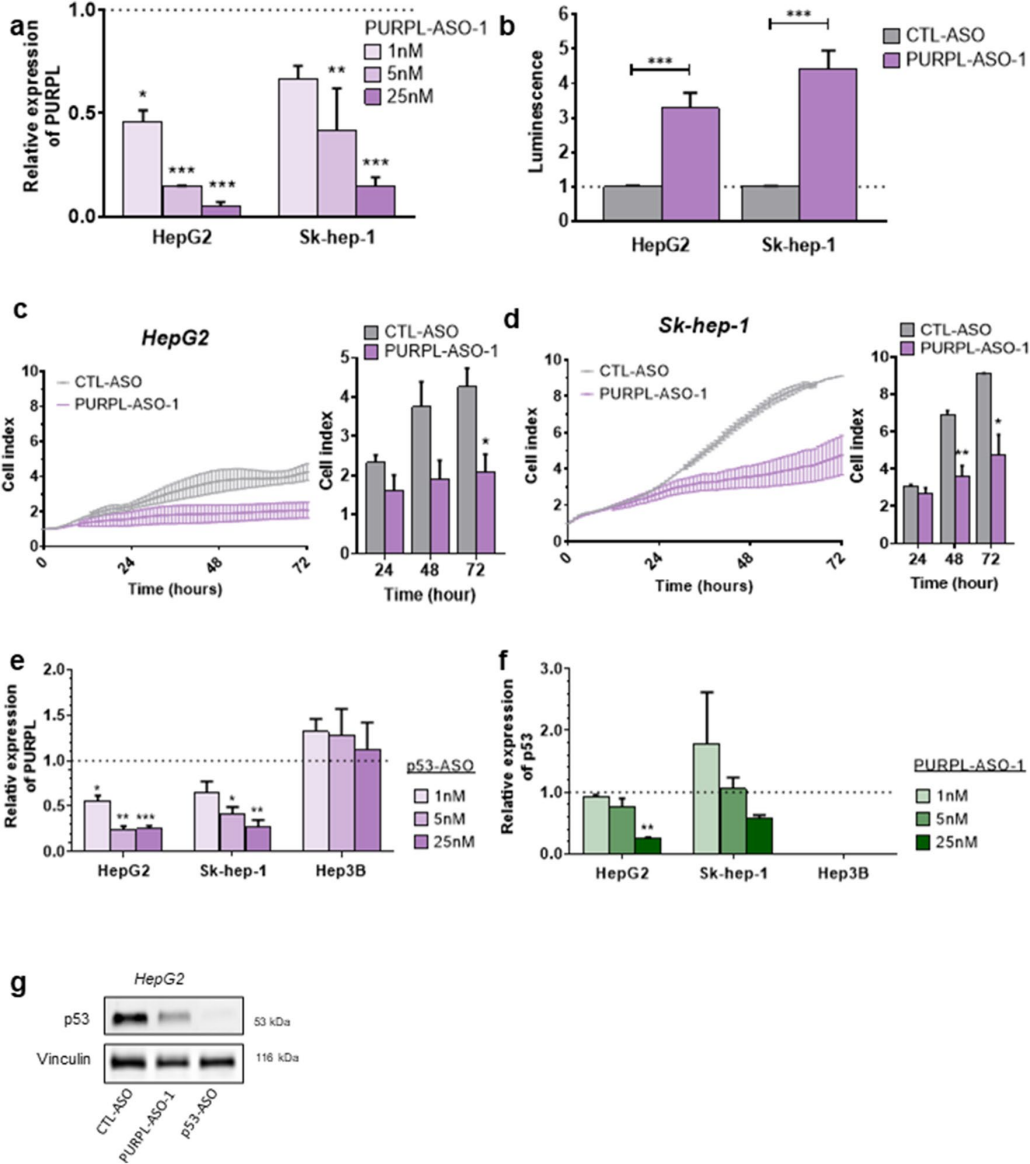
Knockdown of PURPL induces apoptosis and decreases proliferation in liver cancer cell lines. (**a**) Relative expression of PURPL after transfection with PURPL-ASO-1 or CTL-ASO at 1, 5 and 25 nM concentration in HepG2 and Sk-hep-1 cells, respectively. Data were normalized to TBP and scaled to CTL-ASO (*n* = 2). (**b**) Caspase 3/7 activity measured in HepG2 and Sk-hep-1 cells 48 h after transfection with PURPL-ASO-1 at 25 nM concentration. Data were scaled to CTL-ASO (*n* = 3). (**c, d**) Proliferation measured by cellular impedance after transfection with PURPL-ASO-1 or CTL-ASO at 25 nM concentration for 72 h in HepG2 (*n* = 4) and Sk-hep-1 cells (*n* = 3), respectively. The curve depicts cell growth normalized to a time-point immediately after adhesion (normalized cell index). Shown are bar plots of the same data 24, 48 and 72 h after transfection. (**e, f**) Relative expression of PURPL and p53 after transfection with PURPL-ASO-1, p53-ASO, or CTL-ASO at 1, 5 and 25 nM concentration in HepG2, Sk-hep-1, and Hep3B cells, respectively. Data were normalized to TBP and scaled to CTL-ASO at respective concentrations (*n* = 2–4). (**g**) Western blot analysis of p53 protein upon ASO-mediated knockdown with CTL-ASO, PURPL-ASO-1, or p53-ASO at 25 nM. Vinculin was used as loading control. The membrane was cut prior to hybridization with the primary antibody and cropped for publication. See Supplementary Fig. S4 for raw image blots. All data represent mean values ± SEM. *, ** and *** represents *P* < 0.05, *P* < 0.01, and *P* < 0.001, respectively (Two-way student’s t-test).

To investigate loss-of-function of PURPL in liver cancer, we designed three LNA-modified gapmer ASOs targeting *PURPL* (Supplementary Table 3, Supplementary Fig. S2c + d). Two ASOs (PURPL-ASO-1 and PURPL-ASO-3) showed dose-dependent knockdown of *PURPL* in HepG2 and Sk-hep-1 cells (Fig. 2a; Supplementary Fig. S2e), respectively, and these were investigated for hybridization-dependent toxicity in the mouse embryonic fibroblast cell line 3T3-L1^25^. PURPL-ASO-3 exhibited a higher dose-dependent increase in caspase activity (Supplementary Fig. S2f.) compared to PURPL-ASO-1. Hence, PURPL-ASO-1 was selected for further studies. We transfected HepG2 and Sk-hep-1 cells with PURPL-ASO-1 and measured caspase activity. Knockdown of *PURPL* significantly induced caspase activity in HepG2 (*P* < 0.001) and Sk-hep-1 cells (*P* < 0.001) (Fig. 2b). Furthermore, knockdown of *PURPL* significantly decreased proliferation of HepG2 (*P* = 0.02, 72 h) and Sk-hep-1 cells (*P* = 0.006, 48 h; *P* = 0.02, 72 h) (Fig. 2c + d), suggesting that *PURPL* could function as a potential pro-survival gene in liver cancer.

Next, we investigated the interaction between *PURPL* and *p53* in the two p53 wild-type liver cell lines (HepG2 and Sk-hep-1)^26,27^ and the p53 null HCC cell line (Hep3B)^28^. We confirmed the p53 deletion in Hep3B using specific qPCR primers targeting exon 1-2 and 11 of the p53 transcript. The expression of exon 1-2 in p53 was very low in Hep3B compared to HepG2 and Sk-hep-1 (Supplementary Fig. S3a + b), and exon 11 of p53 was undetectable in Hep3B (Supplementary Fig. S3c), which is consistent with the absence of p53 protein in Hep3B, as assessed by western blot analysis (Supplementary Fig. S3c). Transfection of HepG2, Sk-hep-1, and Hep3B cells with an ASO targeting p53 resulted in a dose-dependent knockdown of *p53* (Supplementary Fig. S3d). A decreased expression of *PURPL* upon *p53* knockdown was observed in the HepG2 and Sk-hep-1 cells, but not in Hep3B cells (Fig. 2e). Furthermore, ASO-mediated knockdown of *PURPL* resulted in a dose-dependent decrease of *p53* and *p21* expression in HepG2 and Sk-hep1 (Fig. 2f, Supplementary Fig. S3f) and p53 protein levels (Fig. 2g). Our findings suggest a regulatory feedback mechanism between *PURPL* and *p53,* which is consistent with previously reported findings in CRC^23^.

### *PURPL* is p53-dependent in liver cancer

**Figure 3:**
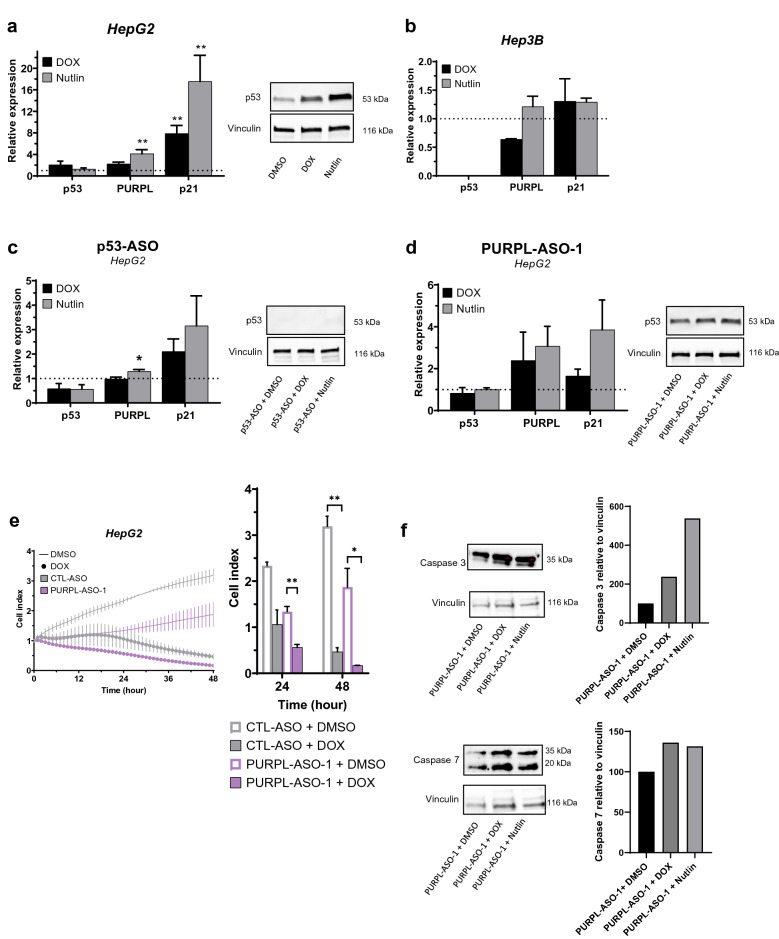
PURPL expression is p53 dependent in liver cancer cell lines. (**a, b**) p53, PURPL and p21 expression assessed by RT-qPCR in HepG2 and Hep3B cells, respectively, treated with doxorubicin (300 nM) or nutlin (3.4 µM). Western blot analysis of p53 protein in HepG2 cells treated with DMSO (0.5%), doxorubicin (300 nM) or nutlin (3.4 µM). Vinculin was used as loading control. The membrane was cut prior to hybridization with the primary antibody and cropped for publication. See Supplementary Fig. S4 for raw image blots. (**c, d**) p53, PURPL and p21 expression assessed by RT-qPCR in HepG2 cells treated with doxorubicin (300 nM) or nutlin (3.4 µM) in combination with p53-ASO or PURPL-ASO-1, respectively, at 25 nM. Data were normalized to TBP, scaled to CTL-ASO and represent two to six biological replicates. Western blot analysis of p53 protein in HepG2 cells treated with DMSO (0.5%), doxorubicin (300 nM), or nutlin (3.4 µM) in combination with PURPL-ASO-1 or p53-ASO (25 nM). Vinculin was used as a loading control. The membrane was cut prior to hybridization with the primary antibody and cropped for publication. See Supplementary Fig. S4 for raw image blots (**e**) Proliferation measured by cellular impedance of HepG2 cells transfected with either CTL-ASO or PURPL-ASO-1 for 24 h before DMSO (0.5%) or doxorubicin (5 µM; *n* = 2) treatment. The curves depict cell growth normalized to the time-point of DMSO or doxorubicin addition (normalized cell index), and the bar plots represent the same data at 24, and 48 h after drug treatment. **(f)** Western blot analysis of caspase-3 and -7 protein in HepG2 cells treated with DMSO (0.5%), doxorubicin (300 nM), or nutlin (3.4 µM) in combination with PURPL-ASO-1 (25 nM). Vinculin was used as a loading control. The membrane was cut prior to hybridization with the primary antibody and cropped for publication. See Supplementary Fig. S4 for raw image blots. All data represent mean values ± SEM. *, ** and *** represents *P* < 0.05, *P* < 0.01, and *P* < 0.001, respectively (Two-way Student’s t-test).

Next, we asked whether enhanced level of endogenous p53 could lead to elevated *PURPL* expression in liver cancer cells. To this end, we used the chemotherapeutic agent DOX that induces a DNA damage response in cells leading to activation of the p53 pathway, and nutlin that stabilizes the p53 protein through binding of MDM2^29,30^. As a control, we analyzed the mRNA and protein levels of *p53* in DOX- and nutlin-treated liver cancer cell lines. The expression of *p53* transcript was unaffected in HepG2 (Fig. 3a) and Sk-hep-1 cells (Supplementary Fig. S3g), but showed elevated p53 protein levels in HepG2 cells (Fig. 3a) upon treatment with either DOX or nutlin. In the same cells, we assessed the expression of *p21*, a downstream target of p53 protein, and observed an increase of *p21* in the p53 wild-type liver cancer cell lines HepG2 (Fig. 3a) and Sk-hep-1 (Supplementary Fig. S3g), but not in the p53 null liver cancer cell line Hep3B (Fig. 3b). This is consistent with the notion that DOX- and nutlin-mediated upregulation of *p21* is dependent on the p53 protein and not the p53 transcript. By comparison, DOX and nutlin treatment of the p53 wild-type liver cancer cell lines HepG2 and Sk-hep-1 also resulted in significant upregulation of *PURPL*, compared to the p53 null liver cancer cell line Hep3B (Fig. 3a + b; Supplementary Fig. S3g). Furthermore, we observed that knockdown of either *p53* or *PURPL* reduced the effect of DOX and nutlin treatment on *PURPL* expression (Fig. 3c + d; Supplementary Fig. S3h + i). However, knockdown of *PURPL* prior to DOX or nutlin treatment did not affect p53 protein levels in HepG2 cells (Fig. 3d), suggesting that DOX- and nutlin-mediated upregulation of p53 is dependent on *PURPL*. Finally, we investigated whether knockdown of *PURPL* affects the cellular response to DOX. To this end, we transfected HepG2 cells with the PURPL-ASO-1 in combination with DOX. Interestingly, cellular impedance data showed that knockdown of *PURPL* sensitized cultured HepG2 cells, but not HEK293 cells, to treatment with DOX compared to the CTL-ASO (Fig. 3e, Supplementary Fig. S3j + k). Furthermore, in a similar experimental setup we observed increased caspase-3 and caspase-7 protein levels in cultured HepG2 cells (Fig. 3f.)

## Discussion

p53 is a master tumor suppressor that plays key roles in cell cycle control, apoptosis, senescence, DNA repair, and change in metabolism and metastasis through the transcriptional regulation of its target genes^31^. More than 3500 direct p53 target genes have been identified in a survey of 13 genome-wide studies^32^. The fact that p53 regulates the expression of numerous genes and cellular pathways is undisputed, however, emerging data imply that it is mainly a direct transcriptional activator and not a repressor^32,33^. p53 mutations or deletions are observed in nearly 50% of all human cancers and approximately 30% of HCC cases are affected by p53 mutations or deletions, underscoring the role of p53 in HCC pathogenesis^34,35^. In this study, we showed that *PURPL* is upregulated in both virus-unrelated HCC and in HCV-associated HCC compared to normal liver tissue, and that its expression correlates with p53 expression in the same tumor samples and is p53-dependent in liver cancer cell lines. Furthermore, we found that the p53 related genes *TP53BP1, TP53BP1, p21/CDKN1A, MDM2*, and *BAX* were upregulated in HCC liver samples compared to control livers. However, this did not reach statical significance, except for TP53BP, which was significantly upregulated in the HCC liver tissue (*P* = 1.59E-02) and HCC-HCV liver tissue (*P* = 0.006), respectively, compared to normal liver samples.

To investigate this interaction further we used ASOs to knock down *PURPL* in liver cancer cells and observed a downregulation of p53 at the mRNA and protein level. Similar results were observed using a p53-ASO, where a dose-dependent downregulation of *PURPL* was detected. Moreover, we demonstrated that an increased level of endogenous p53 protein by induction using either DOX or nutlin leads to upregulation of *PURPL* and *p21*. When the cells were transfected with either a PURPL-ASO-1 or p53-ASO prior to treatment with DOX or nutlin, this effect was diminished. Additionally, knockdown of *PURPL* inhibited cell growth and induced apoptotic activity in liver cancer cells. Finally, ASO-mediated knockdown of PURPL sensitizes HepG2 human HCC cells to treatment with DOX, suggesting that PURPL could serve as a new therapeutic target for reversing doxorubicin resistance in HCC.

PURPL has previously been reported in colorectal, gastric, and liver cancer^22–24,36^, where it was demonstrated that knockdown of *PURPL* inhibited cell proliferation, blocked cell cycle progression, and promoted apoptosis. Furthermore, p53 mRNA and protein levels were downregulated in liver tumor samples and p53 protein levels were upregulated upon knockdown of PURPL in HepG2 and Huh7 liver cancer cells^24^. The authors suggested that PURPL promotes cell proliferation in liver cancer through p53 regulation and thus, could serve as a potential therapeutic target for liver cancer^24^. By comparison, our data show that p53 and p21 are downregulated upon ASO-mediated knockdown of PURPL in the liver cancer cell lines HepG2 and Sk-hep-1. Moreover, we demonstrated that increasing the p53 protein levels by treatment with DOX or nutlin leads to upregulation of *PURPL*. One possible explanation for the observed differences could be due to the different knockdown strategy deployed, as ASOs have been shown to be active in the nucleus, whereas siRNAs exert their activity in the cytoplasm. PURPL is located primarily in the nucleus and to obtain efficient knockdown it is important to use the most adequate strategy. These discrepancies underscore the need for further mechanistic studies to elucidate the exact molecular mechanism by which PURPL and p53 interact in liver cancer.

A previous study reported that loss of PURPL sensitizes CRC cells towards the chemotherapeutic drugs DOX and 5-FU, suggesting that PURPL is involved in the p53-mediated response to DNA damage. However, modulating PURPL expression did not affect p53 protein levels or transcriptional activity, implying that PURPL does not form a feedback loop with p53 in CRC, but rather may act as a downstream mediator of p53 function without directly affecting p53 activity^22^. In another study, PURPL was shown to suppress basal p53 levels and promote CRC tumorigenicity, where MYBBP1A was identified as a PURPL-interacting protein through RNA pull-down studies^23^. These conflicting results in HCC and CRC illustrate how complex PURPL-p53 interactions are, and further studies are needed to elucidate these discrepancies.

In summary, our data show that PURPL is upregulated in liver cancer and that *PURPL* and *p53* expression is codependent. Moreover, knockdown of PURPL inhibits cell proliferation and induces apoptosis independent of p53 in response to DNA-damage. Finally, ASO-mediated knockdown of PURPL sensitizes HepG2 human HCC cells to treatment with DOX, suggesting that PURPL could serve as a new therapeutic target for reversing DOX resistance in HCC. Resistance to chemotherapy is a major problem in all cancers, including HCC resulting in relapse of the disease providing a massive setback to therapeutic regimens targeting HCC. Moreover, due to liver dysfunction, liver cancer patients generally have a poor tolerance to chemotherapy. Thus, identification of new molecular targets such as PURPL described in this study, and subsequent characterization of the cellular mechanisms underlying chemoresistance are urgently needed to develop new therapeutic strategies for effectively reversing chemoresistance in HCC.

## Materials and methods

### Cell lines

The human liver cancer cell lines Hep3B, HepG2, and Sk-hep-1 were purchased from the European Collection of Authenticated Cell Cultures (ECACC, UK). The mouse embryonic fibroblast cell line 3T3-L1 and normal liver cell line Thle-3 were acquired from the American Tissue Culture Collection (ATCC). The embryonic kidney cell line HEK293 was purchased from Sigma. Hep3B and HepG2 cells were cultured in Eagles Minimum Essential Medium (EMEM; Sigma) supplemented with 10% fetal bovine serum (FBS; Sigma), 1% Glutamine (2 mM; Sigma), 1% Penicillin–Streptomycin antibiotic (P/S; Sigma) and 1% Non-Essential Amino acid (NEAA; Sigma). Sk-Hep-1 cells were cultured in EMEM (Sigma) supplemented with 10% FBS (Sigma), 1% Glutamine (2 mM; Sigma), 1% P/S (Sigma), 1% NEAA (Sigma) and 1% sodium pyruvate solution (1 mM, sterile-filtered, Sigma). The 3T3-L1 and HEK293 cell lines were cultured in Dulbecco’s Modified Eagle Medium (DMEM; Sigma) supplemented with 10% FBS (Sigma), 1% Glutamine (2 μM; Sigma) and 1% P/S (Sigma). Thle-3 cell line was cultured in Bronchial Epithelial Cell Growth Media (BEGM) bulletkit (#CC-3170; Lonza), which includes 500 mL basal medium and separate frozen additives from which we discard the gentamycin/ Amphotericin (GA) and Epinephrine supplemented with 10% FBS (Sigma), 5 ng/mL epidermal growth factor (EGF; E9644-0.2MG; Merck), 70 ng Phosphoethanolamine (P0503-1G; Merck) and 1% P/S (Sigma). All cell cultures were incubated in 5% CO^2^ at 37 °C. Cell counts were performed with Countess II FL Automated Cell Counter (Thermo Fisher Scientific).

### RNA extraction

Total RNA from cells was isolated using the RNeasy kit (Qiagen) according to the manufacturer’s recommendations. Subcellular separation of nuclear and cytoplasmic RNA fractions was carried out using the PARIS Kit (Applied Bioscience). The RNA concentration was measured using a microplate reader (Varioscan LUX multimode microplate reader, Thermo Scientific) at 260 nm.

### Liver biopsies

Sections of formalin-fixed paraffin embedded (FFPE) archival human liver biopsies from 24 patients were obtained from Department of Pathology, Aalborg University Hospital, Aalborg, Denmark. The biopsies included ten HCV-HCC, ten HCC, and four normal liver samples. This study has been approved by the Danish National Committee on Health Research Ethics (nr. 1,703,994) and in accordance with the Declaration of Helsinki. Informed consent was obtained from all subjects and/or their legal guardian(s).

Total RNA from liver biopsies was purified using RNeasy FFPE Kit (Qiagen) according to the manufacturer’s instructions. RNA quality was assessed using the Experion automated electrophoresis system (BioRad).

### RNA sequencing

Total RNA libraries were prepared by Center for Genomic Medicine at Rigshospitalet, using the NEBNext Ultra RNA Library Prep Kit (Illumina). The input for each sample was 100 ng total RNA, except for sample 22 and 23 where 50 ng were used. RNAseq was performed on HiSeq2500 using a HiSeq SBS Kit v4 250 cycle (2 × 125 bp paired end sequencing), providing un-stranded paired-end raw reads in FASTQ format.

### Total RNAseq data analysis

Spliced Transcripts Alignment to a Reference (STAR) aligner (v. 2.5.0a) was used for read mapping and gene quantification using Ensembl release 92 of *Homo sapiens* as reference. To account for fragment amplification during library preparation, mean gene count values were assessed across each disease group. Genes with a mean expression of at least 5 in 70% of the samples, in one or more disease groups were included for further analysis. Gene count matrices for each disease group were imported in R (v. 3.6.1) and principal component analysis was calculated using the *prcomp* package. The DESeq2 package (v. 1.24.0) was used for count normalization and differential expression analysis. Normalized count values from significantly differentially expressed genes (α_FDR_ < 0.05 and log2 fold change > 1), were selected for hierarchical clustering and visualized using the pheatmap (v. 1.0.12) package^37^. Principal component analysis and normalized counts for PURPL and p53-regulated genes were visualized using the ggplot2 R package (version 3.2.1)^38^. Significant differentially expressed genes were visualized in volcano plots using the EnhancedVolcano R package (version 1.12.0)^39^.

### Quantitative reverse transcription PCR (qRT-PCR)

Isolated total RNA was reverse transcribed using the QuantiNova Reverse Transcription Kit (Qiagen). Quantitative Reverse Transcription PCR (qRT-PCR) was performed using FAM-labelled pre- and custom designed PrimeTime qPCR probe assays (IDT) (Supplementary Table S2) and QuantiNova Probe PCR Kit (Qiagen) measured on the Quant-Studio 6 Flex Real-Time PCR system (Applied Biosystems).

### LNA-modified gapmer antisense oligonucleotides

The oligonucleotides used in this study are listed in Table S3 and were synthesized as unconjugated LNA-modified DNA oligonucleotides with a complete phosphorothioate backbone (Qiagen).

### Knockdown experiments

Cultured cells were seeded out one day prior to transfection to allow the cells to adhere. After 24 h the cells were washed once in Opti-MEM (Gibco) and transfected using a mixture of Lipofectamine 2000 (5 µg/mL (Sk-Hep-1), 10 µg/mL (Hep3B), 10 µg/mL (HEK293) 10 µg/mL (THLE-3) ThermoFisher), Opti-MEM and the ASOs at a final concentration of 1, 5 or 25 nM. Four hours after transfection, the mixture was carefully removed from the cells and fresh medium was added to each well. HepG2 cells were reverse-transfected. A mixture of Lipofectamine 2000 (2.5 µg/mL, ThermoFisher), Opti-MEM combined with the ASOs at a final concentration of 1, 5 or 25 nM were incubated at RT, and the HepG2 cells were added after 15 min of incubation. Knockdown was assessed 48 h after transfection. The cells were harvested, total RNA isolated and RNA expression measured using RT-qPCR as described.

In the experiments in which the effect of PURPL knockdown was assesses in combination with Doxorubicin or Nutlin treatment, cells were transfected as described above with ASOs at a final concentration of 25 nM for 24 h before DMSO (0.5%), Doxorubicin (Sigma; 5 µM) or Nutlin (Sigma; 5 µM) was added to the appropriate wells. Both Doxorubin and Nutlin were reconstituted in DMSO (Sigma).

### Western blot analysis

Cells were homogenized in RIPA buffer supplemented with cOmplete, Mini, EDTA-free Protease Inhibitor Tablets (Sigma), resuspended using a syringe and centrifuged for 30 min at 4 °C. The protein concentration was determined using the DC Protein Assay (Bio-Rad) according to the manufacturer’s instruction. 25 µg whole cell lysate was loaded per lane in 4–15% gradient agarose gels (Bio-Rad) and transferred onto 0.2 µm nitrocellulose membranes (Bio-Rad). The membranes were blocked in 5% skim milk for 1 h, cut in two, and then probed with a primary antibody for p53 (DO-1; SC-47698, 1:100; Santa Cruz), caspase-3 (9662, 1:250; Cell Signaling), or caspase-7 (9492, 1:250; Cell Signaling) on one-half of the blot, or vinculin control (V9131-0.2ML; 1:5000; Sigma) on the other half of the membrane overnight at 4 °C. Overnight incubation was followed by goat anti-mouse secondary HRP antibody (1:20,000, Dako) incubation for 1 h at room temperature. Visualization was achieved with SuperSignal West Pico PLUS Chemiluminescent Substrate (#34577; Thermo Fisher) on the ChemiDoc Touch Imaging System (Bio-Rad) with the setting optimal rapid exposure. Since the membranes were cut prior to hybridization with antibodies the full-length blots cannot be provided. Image Studio Lite (Licor) was deployed for protein quantification of caspase-3 and caspase-7 relative to vinculin control.

### Cell apoptosis assay

Caspase activity was analyzed using the Caspase-Glo 3/7 Assay (Promega) according to the manufacturer’s instructions. The reagents were added to each well 48 h after transfection and luminescence was measured one hour after addition of reagents using the Varioskan LUX multimode microplate reader (Thermo Fischer Scientific).

### Real-time impedance measurement

Real-time analysis of proliferation was performed using the xCELLigence RTCA DP instrument (ACEA Biosciences) which was placed in an incubator at 37 °C and 5% CO_2_ atmosphere. Cultured cells were transfected, as described above, in disposable E-plates. The noninvasive gold electrodes covering 80% of the well, quantified cell proliferation and changes in morphology based on impedance.

### PURPL expression and survival analysis in the TCGA/GTex database

PURPL expression data in The Cancer Genome Atlas (TCGA) and Genotype-Tissue Expression (GTex) databases were accessed through the online platform MiPanda (http://www.mipanda.org/)^40^ to assess PURPL expression in liver a tumor data set (*n* = 556).

### Statistical analysis

Results are reported as mean ± standard error of the mean (SEM) of 2–5 biological replicates where each biological replicate comprises 2–4 technical replicates. Significance was determined using a two-way Student’s t-test. Differences were considered significant at *P* < 0.05.

## Supplementary Information

Supplementary Information 1.

Supplementary Information 2.

Supplementary Information 3.

Supplementary Information 4.

## Data Availability

The data have been uploaded to NCBI (accession numbers GSE185700) and will be released upon publication.
