# Supplementary material for: Knockdown of the long noncoding RNA PURPL induces apoptosis and sensitizes liver cancer cells to doxorubicin
Source: Sci Rep. 2022 Nov 14;12:19502. doi: 10.1038/s41598-022-23802-9 (PMC9663437; doi:10.1038/s41598-022-23802-9)
Supplement: Supplementary file 1 — Supplementary Information 1. [file 41598_2022_23802_MOESM1_ESM.docx]

Knockdown of the long noncoding RNA PURPL induces apoptosis and sensitizes liver cancer cells to doxorubicin

Tsinat Berhane^1#,§^, Anja Holm^1#^, Kasper Thystrup Karstensen^1¤^, Andreas Petri^1,Ɨ^, Mirolyuba Simeonova Ilieva^1^, Henrik Krarup^2^, Mogens Vyberg^1,3^, Marianne Bengtson Løvendorf^1£^ and Sakari Kauppinen^1*^

^1^Center for RNA Medicine, Department of Clinical Medicine, Aalborg University, Copenhagen, Denmark

^2^Department of Molecular Diagnostics, Aalborg University Hospital, and Department of Clinical Medicine, Aalborg University, Aalborg, Denmark

^3^Department of Pathology, Aalborg University Hospital, and Department of Clinical Medicine, Aalborg University, Aalborg, Denmark

§Present address: Department of Health Technology, Technical University of Denmark, Kgs. Lyngby, Denmark

¤Present address: Department of Bacteria, Parasites and Fungi, Statens Serum Institute, Copenhagen, Denmark

^£^Present address: Department of Dermatology and Allergy, Herlev and Gentofte Hospital, University of Copenhagen, Hellerup, Denmark

^Ɨ^Present address: Department of Bioinformatics, Lundbeck, Valby, Denmark

^#^Equal contribution

*Corresponding author:

E-mail: ska@dcm.aau.dk

# LEGENDS TO SUPPLEMENTARY FIGURES

**Figure S1.** Volcano plots and expression profiling of HCC, HCV, and normal liver samples. **(a)** Volcano plots of expression levels for all genes in HCC relative to normal liver biopsies samples. The horizontal line highlights the *P* value cutoff of 0.05 converted to a -log10 scale. The vertical line highlights the log2 fold change (Log2FC) cutoffs. Purple dots denote significantly differentially expressed genes with an unadjusted *P* value < 0.05 and FC<1.5 (-log10[p value], log2 fold change, respectively) that were included in the study. Green dots denote genes with FC<1.5 and *P* > 0.05 (-log10[p value], log2 fold change, respectively), blue dots denote genes with FC>1.5 and *P* < 0.05 (-log10[p value], log2 fold change, respectively), and black dots denote genes *P* > 0.05 and FC>1.5 (-log10[p value], log2 fold change, respectively). **(b)** Volcano plots of expression levels for all genes in HCV-HCC relative to normal liver biopsies samples. The horizontal line highlights the *P* value cutoff of 0.05 converted to a -log10 scale. The vertical line highlights the log2 fold change (Log2FC) cutoffs. Purple dots denote significantly differentially expressed genes with an unadjusted *P* value < 0.05 and FC<1.5 (-log10[p value], log2 fold change, respectively) that were included in the study. Green dots denote genes with FC<1.5 and *P* > 0.05 (-log10[p value], log2 fold change, respectively), blue dots denote genes with FC>1.5 and *P* < 0.05 (-log10[p value], log2 fold change, respectively), and black dots denote genes *P* > 0.05 and FC>1.5 (-log10[p value], log2 fold change, respectively). **(c)** Correlation plot of normalized counts from Purpl and p53 in the RNA seq. (r = 0.4065, *P*=0.0487) dataset **(d-i)** Normalized expression of p53-regulated genes in normal liver samples: **(d)**T-test between TP53 expression in normal liver tissue (*n* = 4) and each of the HCC groups showed a 0.15 and -0.03 (log2) fold increased expression of TP53 in the HCC (*n* = 10; *P* =0.94) and the HCC-HCV groups (*n* = 10; *P* = 0.97). **(e)** T-test between TP53BP2 expression in normal liver tissue (*n* = 4) and each of the HCC groups showed a 1.81 and 1.84 (log2) fold increased expression of TP53BP2 in the HCC (*n* = 10; *P* = 1.59E-02) and the HCC-HCV groups (*n* = 10; *P* = 0.006). **(f)** T-test between TP53BP1 expression in normal liver tissue (*n* = 4) and each of the HCC groups showed a 0.89 and 1.02 (log2) fold increased expression of TP53BP1 in the HCC (*n* = 10; *P* = 0.10) and the HCC-HCV groups (*n* = 10; *P* = 0.03), respectively. **(g)** T-test between CDKN1A/p21 expression in normal liver tissue (*n* = 4) and each of the HCC groups showed a 0.97 and 1.04 (log2) fold increased expression of CDKN1A/p21 in the HCC (*n* = 10; *P* = 0.46) and the HCC-HCV groups (*n* = 10; *P* = 0.29). **(h)** T-test between BAX expression in normal liver tissue (*n* = 4) and each of the HCC groups showed a 0.91 and 0.85 (log2) fold increased expression of BAX in the HCC (*n* = 10; *P* = 0.54) and the HCC-HCV groups (*n* = 10; *P* = 0.56). **(i)** T-test between MDM2 expression in normal liver tissue (n = 4) and each of the HCC groups showed a 0.44 and 1.04 (log2) fold increased expression of MDM2 in the HCC (*n* = 10; *P* = 0.7) and the HCC-HCV groups (*n* = 10; *P* = 0.07).

**Figure S2.** Screen for ASOs targeting PURPL.

(**a**) PURPL expression was assessed by RT-qPCR in HCC cell lines HepG2 and Sk-hep-1 scaled to PURPL expression levels in Thle-3. The y-axis represents fold-change. (**b**) Relative expression levels of PURPL using RT-qPCR of the nuclear and cytoplasmic fractions of untreated Sk-hep-1 cells. GAPDH and MALAT1 were used as controls for the purity of the isolated cytoplasmic and nuclear fractions, respectively. (**c-d**) Relative expression of PURPL 48 hrs after transfection with PURPL-ASO-1, PURPL-ASO-2, PURPL-ASO-3, and CTL-ASO at 25 nM concentration in HepG2 and Sk-hep-1 cells, respectively. Data were normalized to TBP and scaled to CTL-ASO. (**e**) Relative expression of PURPL after transfection with Purpl-ASO-3 and CTL-ASO at 1, 5 and 25 nM concentration in HepG2 and Sk-hep-1 cells, respectively. Data were normalized to TBP and scaled to CTL-ASO (n = 2). (**f**) Caspase 3/7 activity measured in mouse 3T3-L1 fibroblasts cells 48h after transfection with PURPL-ASO-1 and PURPL-ASO-3 at 5, 25, and 50 nM concentration. The data were scaled to CTL-ASO at corresponding concentrations and representative of two biological replicates.

**Figure S3.** p53 expression profile in liver cancer cell lines.

(**a**) RNAseq data expression of p53 in Hep3B, HepG2, and Sk-hep-1 cells expressed in RPM (left panel). (**b-c**) RT-qPCR measurement of p53 expression for exon 1-2 and 11, respectively, in Thle-3, Hep3B, HepG2, and Sk-hep-1. Data were normalized to TBP and scaled to PURPL expression levels in Thle-3. The y-axis represents fold-change. Western blot analysis of p53 protein in Hep3B (15µg and 25µg loaded protein. Vinculin was used as loading control. The membrane was cut prior to hybridization with the primary antibody and cropped for publication. See Supplementary Fig. S4 for raw image blots. (**d-e**) RT-qPCR measurement of p53 expression for exon 1-2 and 11, respectively, 48 hrs after transfection with p53 targeting ASO in Hep3B, HepG2, and Sk-hep-1 (n = 1-2). Data were normalized to TBP and scaled to CTL-ASO. The y-axis represents fold-change. (**f)** RT-qPCR analysis of p21 expression, 48 hrs after transfection with PURPL-ASO-1 or p53-ASO at 25 nM concentration in HepG2 (n = 1-2). Data were normalized to TBP and scaled to CTL-ASO. The y-axis represents fold-change. (**g-i**) p53, PURPL and p21 expression assessed by RT-qPCR in Sk-hep-1 cells treated with doxorubicin (300nM), nutlin (3.4µM) or mentioned drugs in combination with p53-ASO or PURPL-ASO-1, respectively, at 25 nM. Data was normalized to TBP, scaled to CTL-ASO and represent three-seven biological replicates. **(j)** Proliferation measured by cellular impedance of HepG2 cells transfected with either CTL-ASO or PURPL-ASO-1 for 24 hrs before DMSO (0.5%; *n* = 2) or doxorubicin (0.3µM, 2.5µM, or 5µM; *n* = 2) treatment. The curves depict cell growth normalized to the time-point of DMSO or doxorubicin addition (normalized cell index). **(k)** Proliferation measured by cellular impedance of HEK293 cells transfected with either CTL-ASO or PURPL-ASO-1 for 24 hrs before DMSO (0.5%; *n* = 2) or doxorubicin (2.5µM or 5µM; *n* = 3) treatment. The curves depict cell growth normalized to the time-point of DMSO or doxorubicin addition (normalized cell index).

All data represent mean values ± SEM. *, **, *** and *** represents *P* < 0.05, *P* < 0.01, *P* < 0.001, *P* < 0.0001, respectively (Two-way Student’s t-test).

**Figure S4.** Full-length blots.

**Table S1.** List of significant differentially expressed lncRNAs in the HCV-HCC and HCC diagnostic groups relative to the normal liver biopsy samples (*P* < 0.05, FC>1.5).

**Table S2.** List of PCR primers and probes used to detect lncRNA expression.

**Table S3.** LNA-modified ASOs used in this study.

Supplementary figure 1.

Differentially expressed genes in HCC

**a**

**
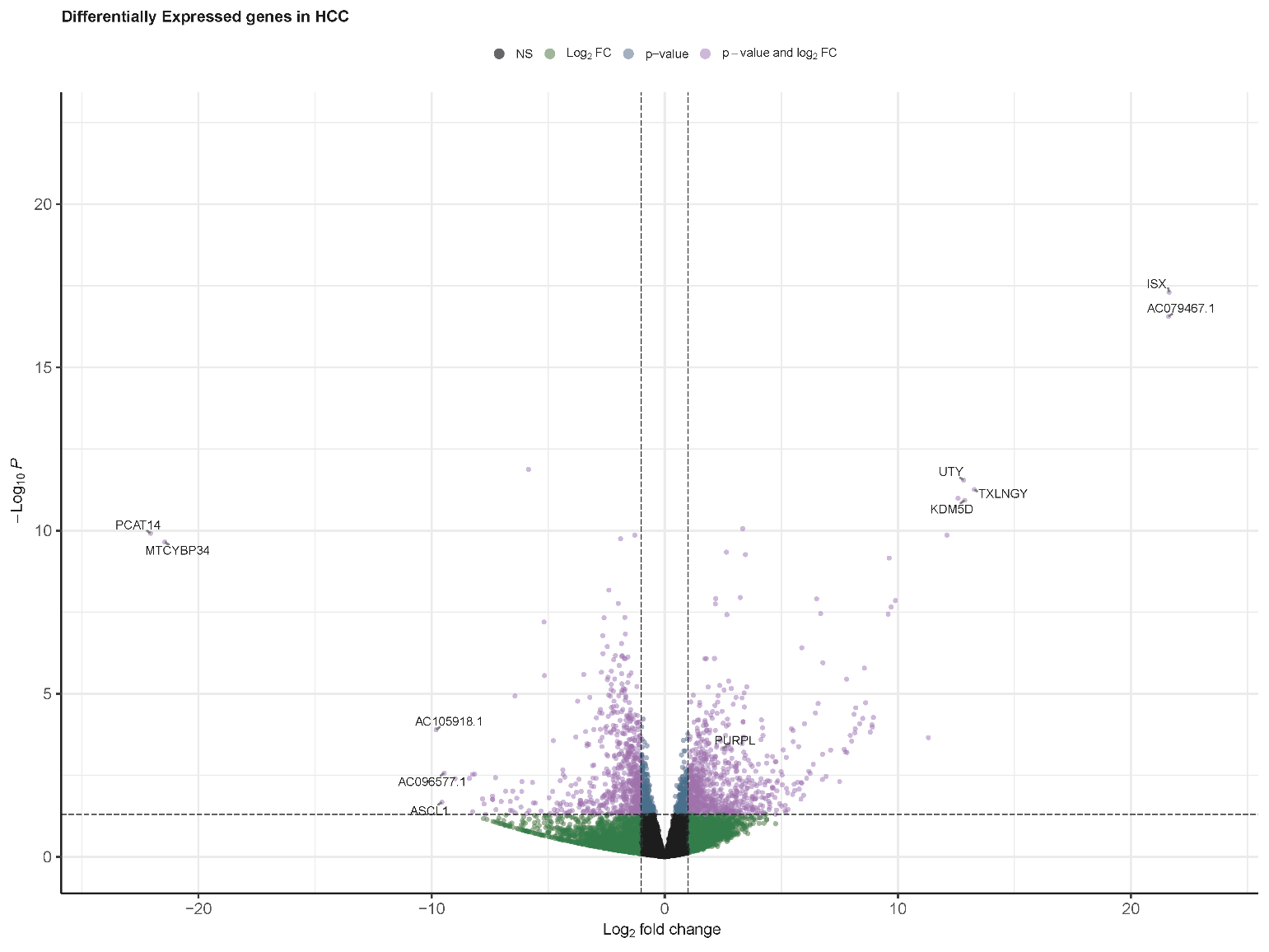

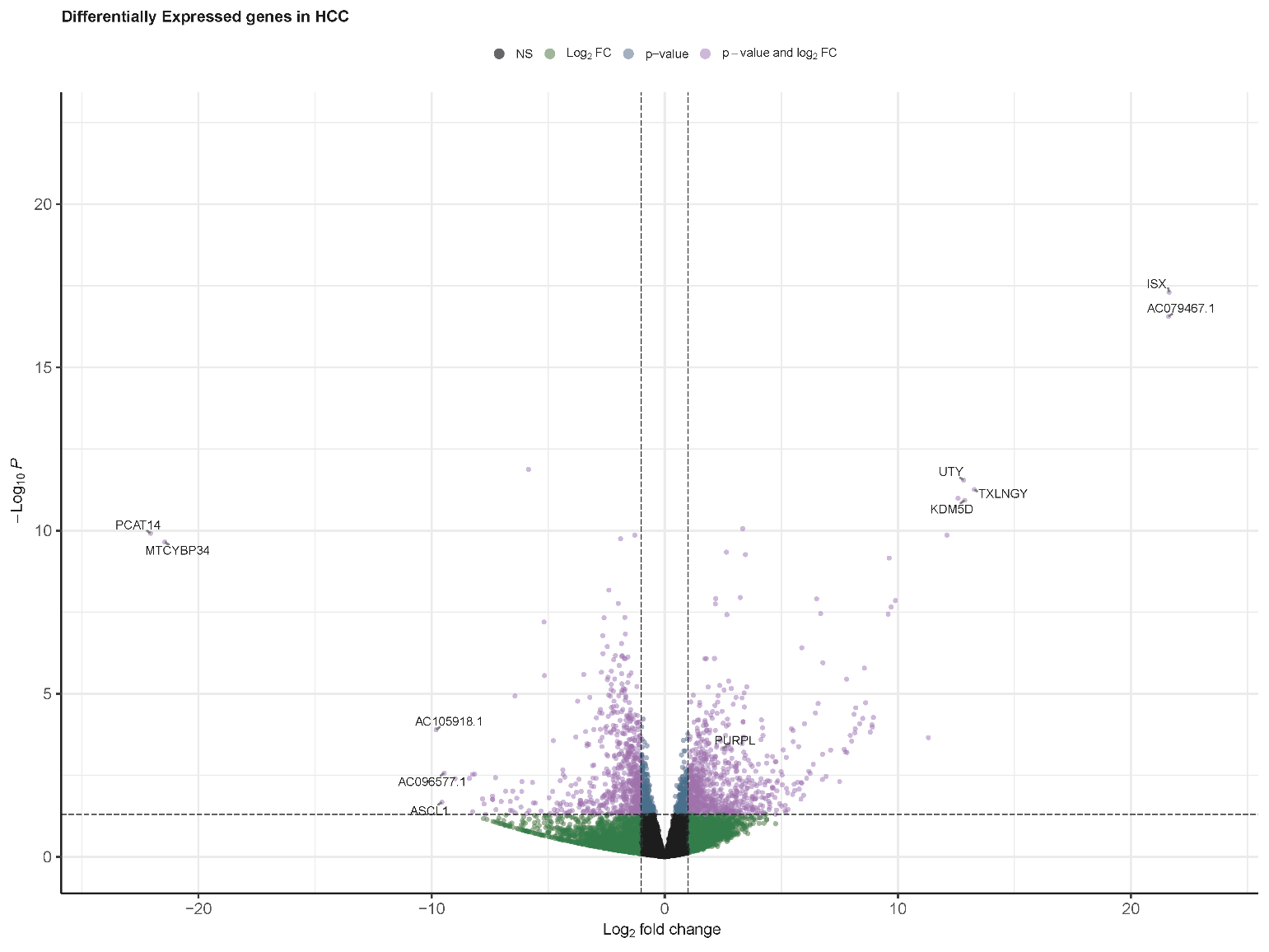
**

**
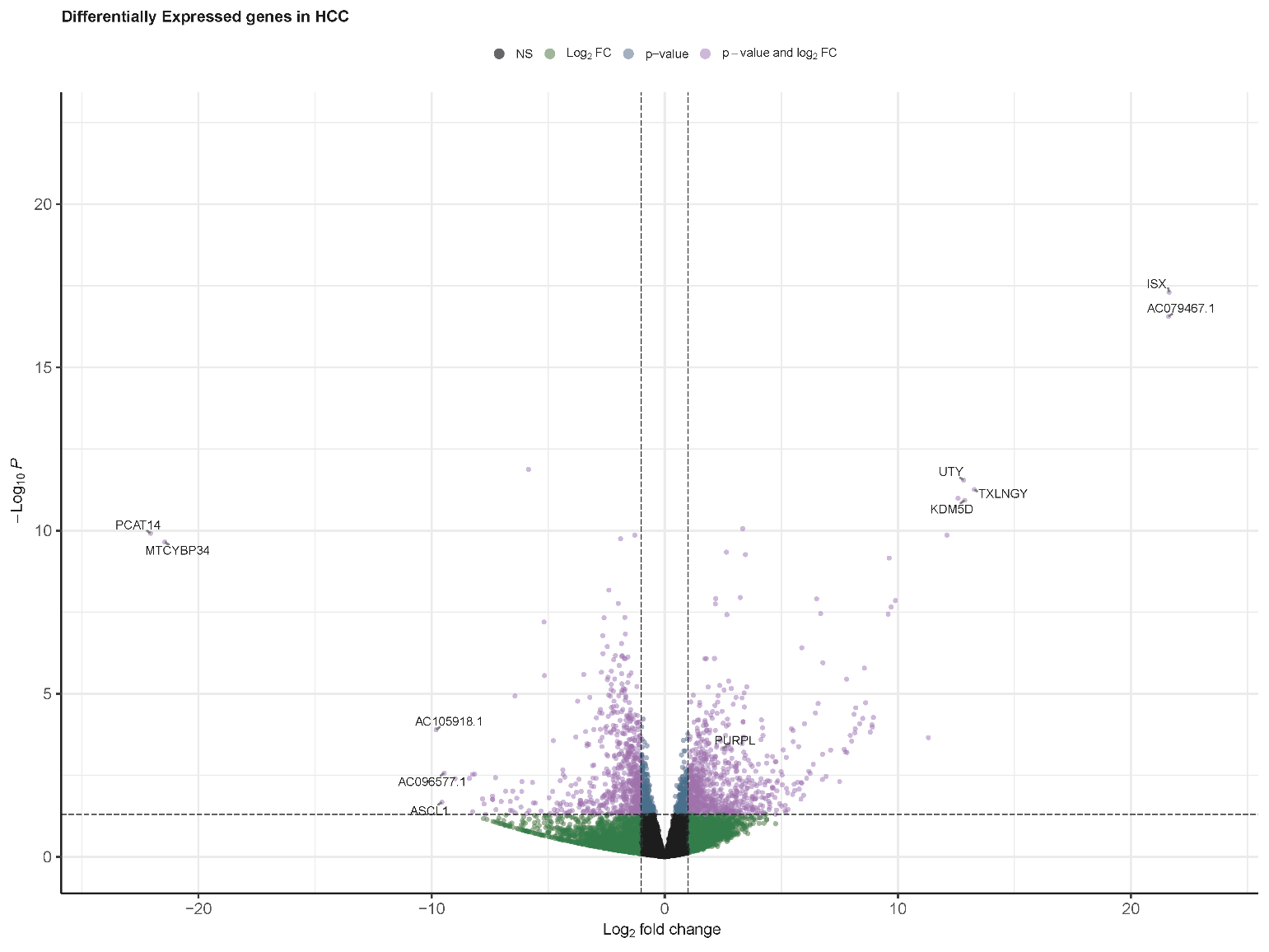
**

**
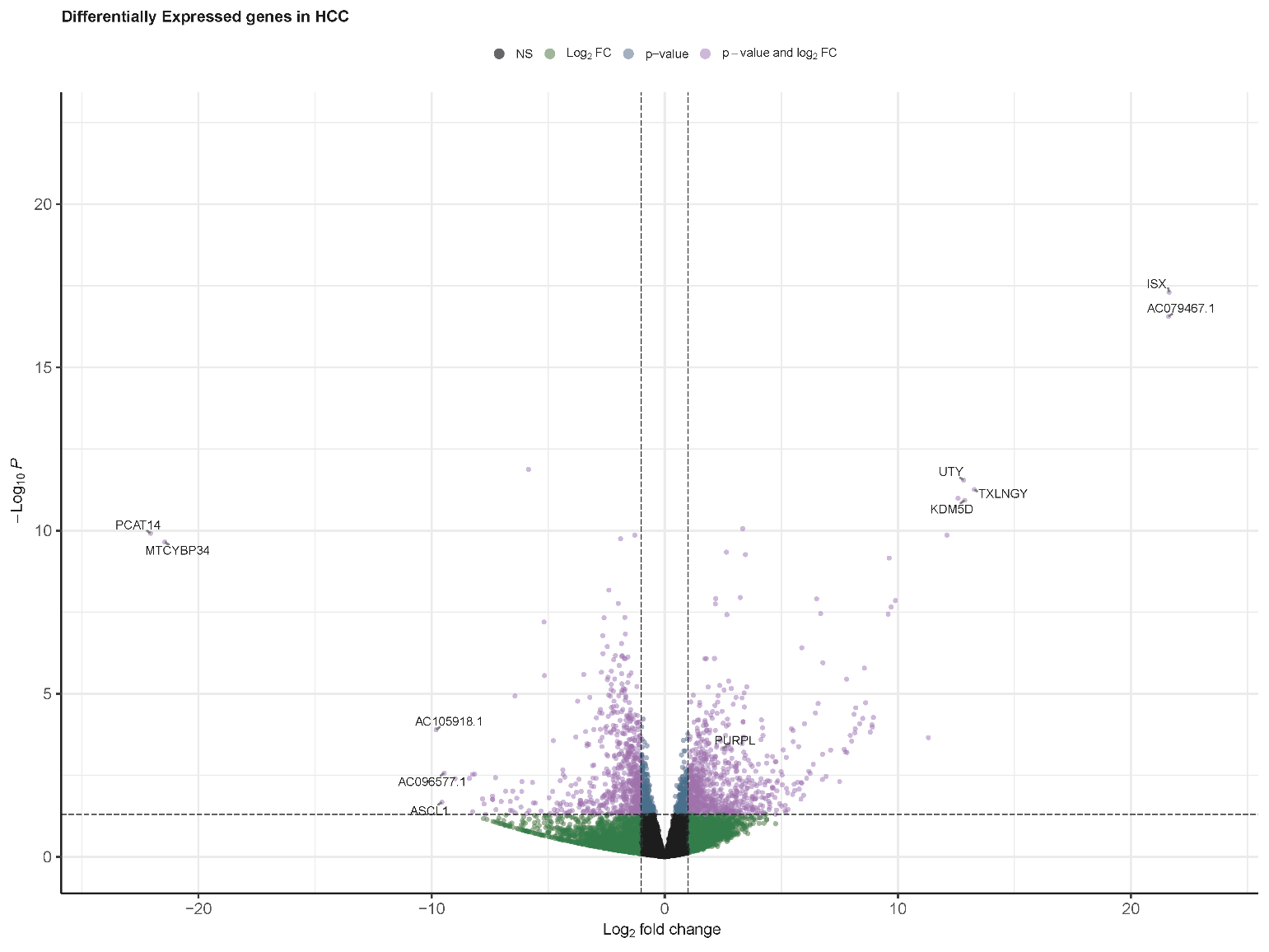
**

Supplementary figure 1 - continue.

Differentially expressed genes in HCV-HCC

**b**

**
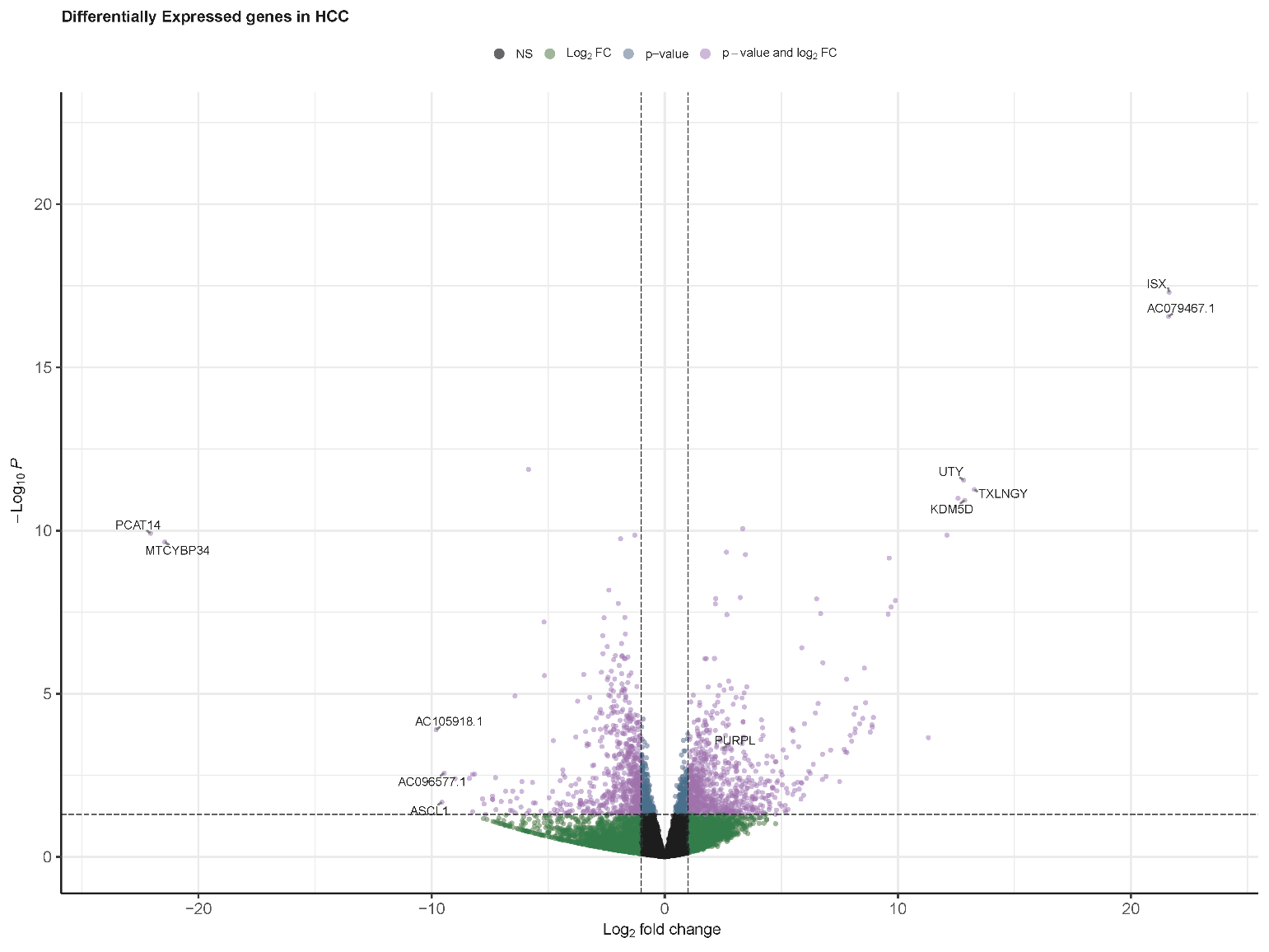
**
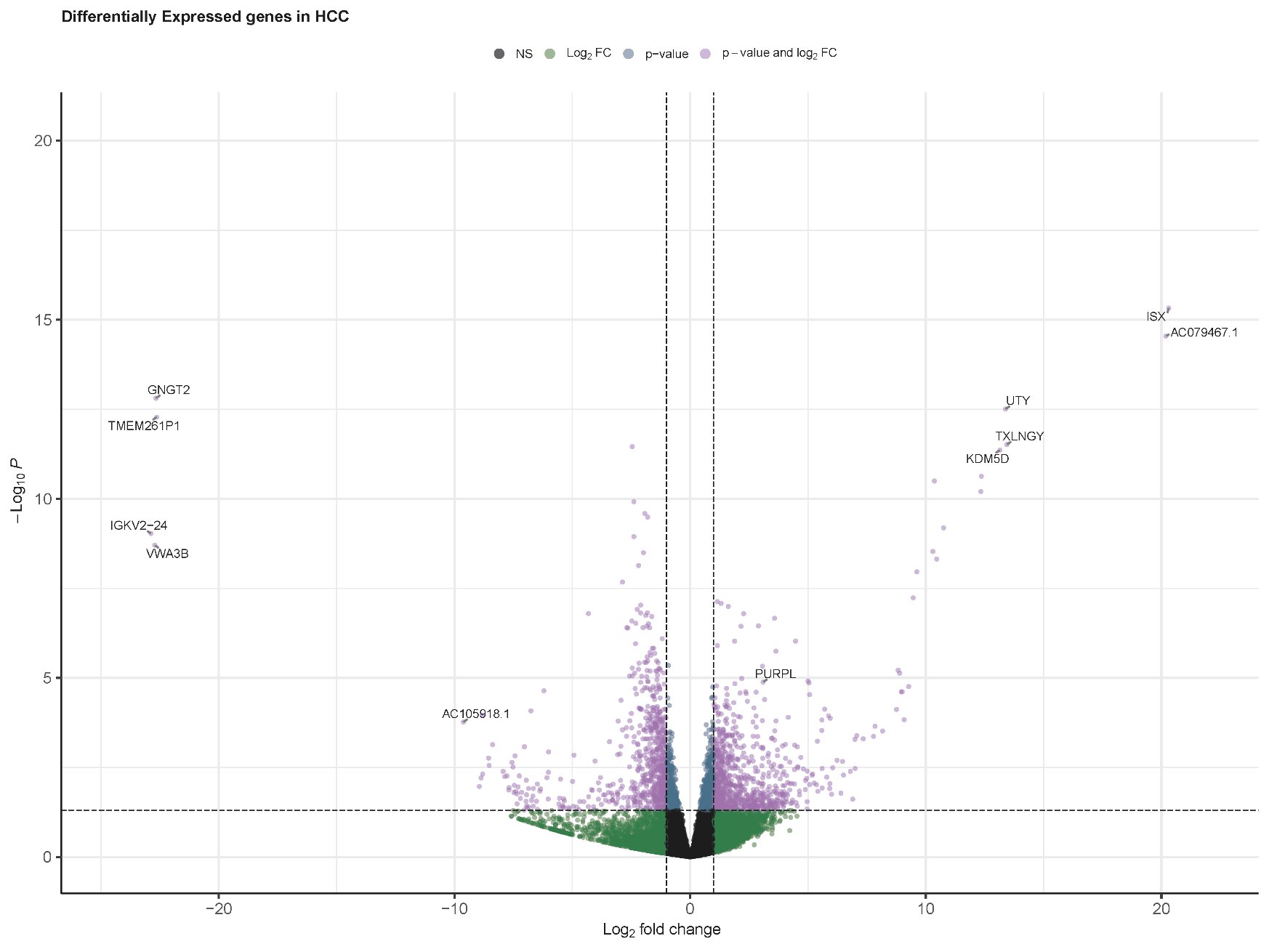


**
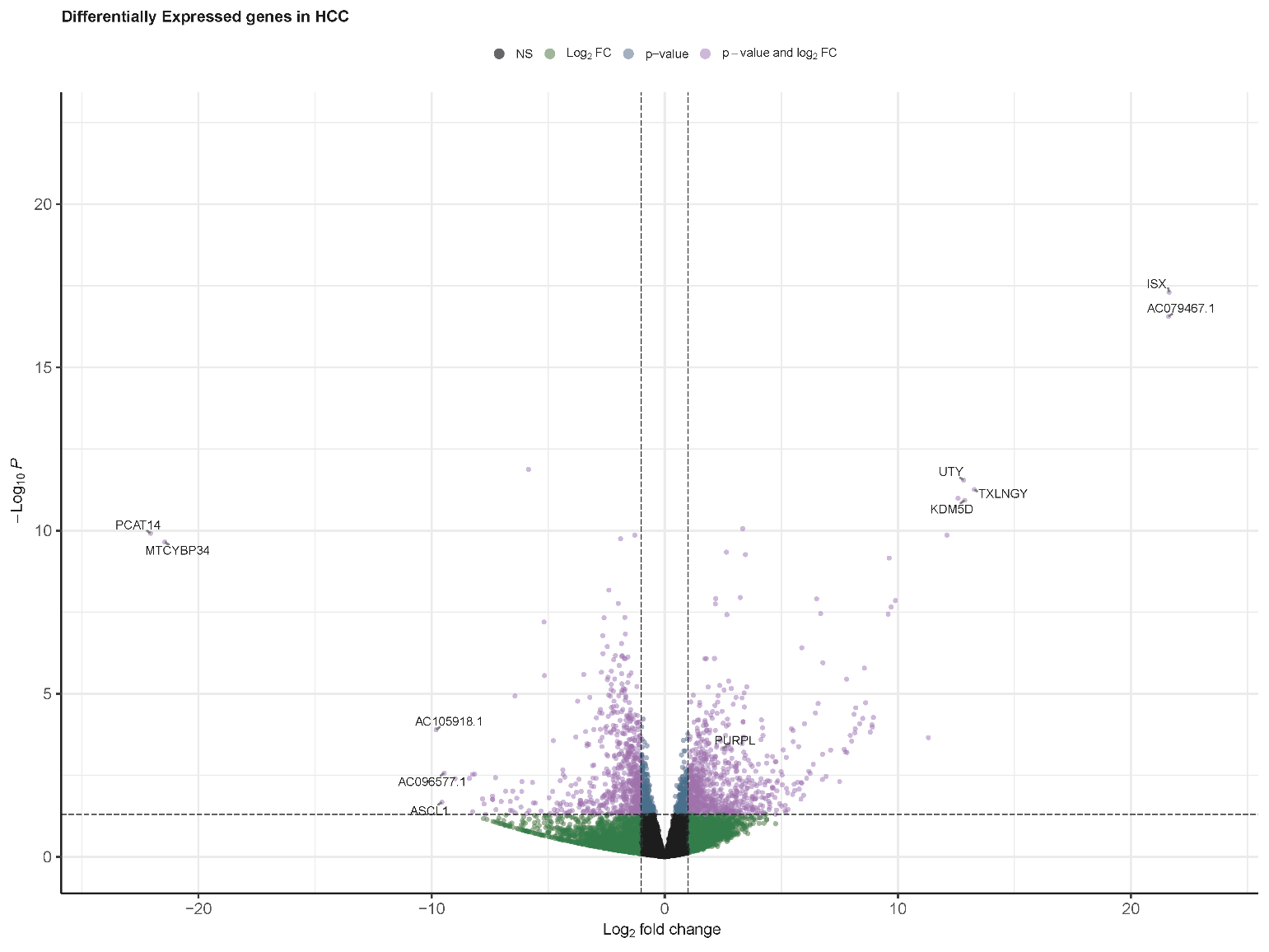
**

**
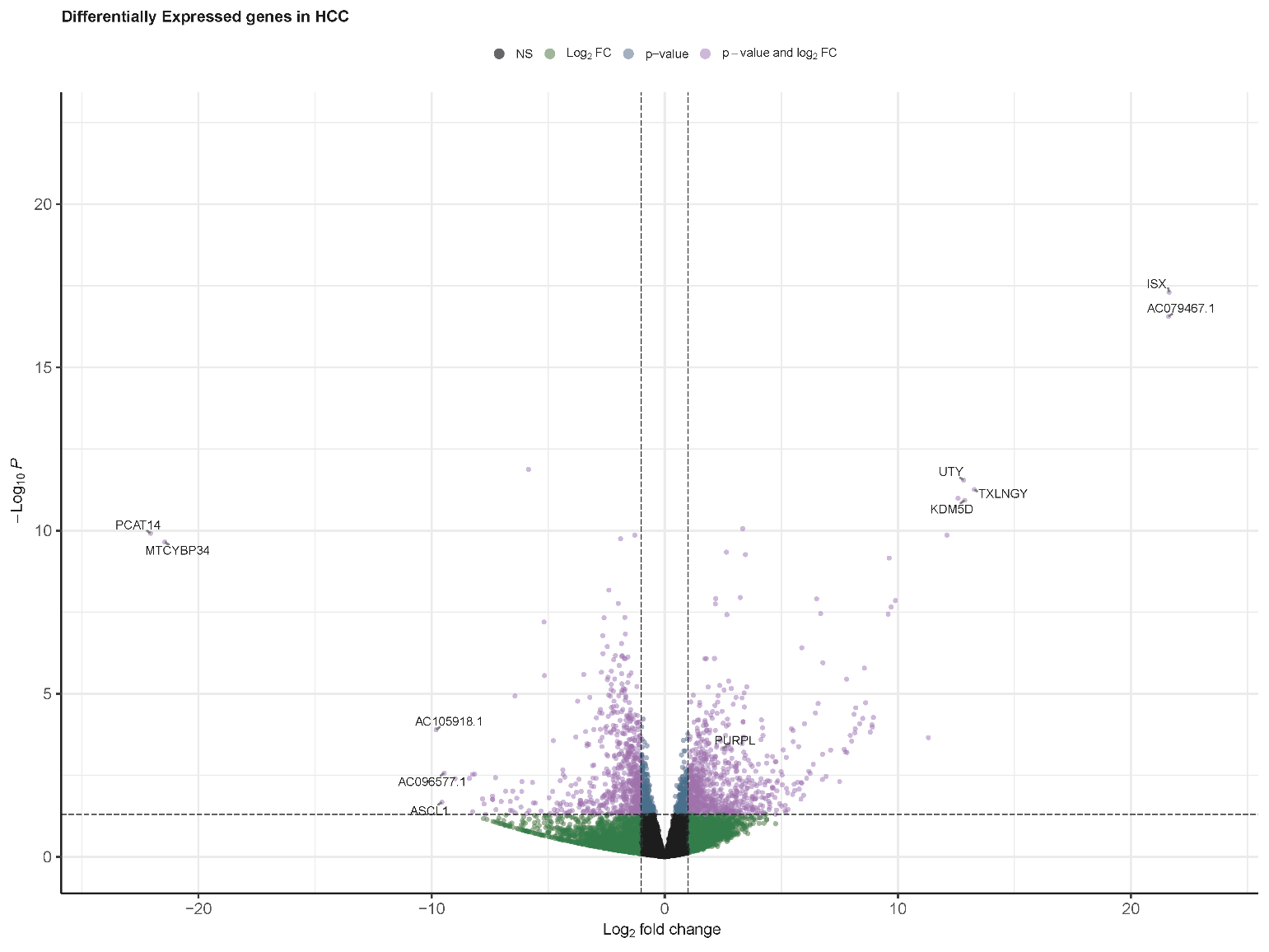
**

**c**

Supplementary figure 1 - continue.

**d**


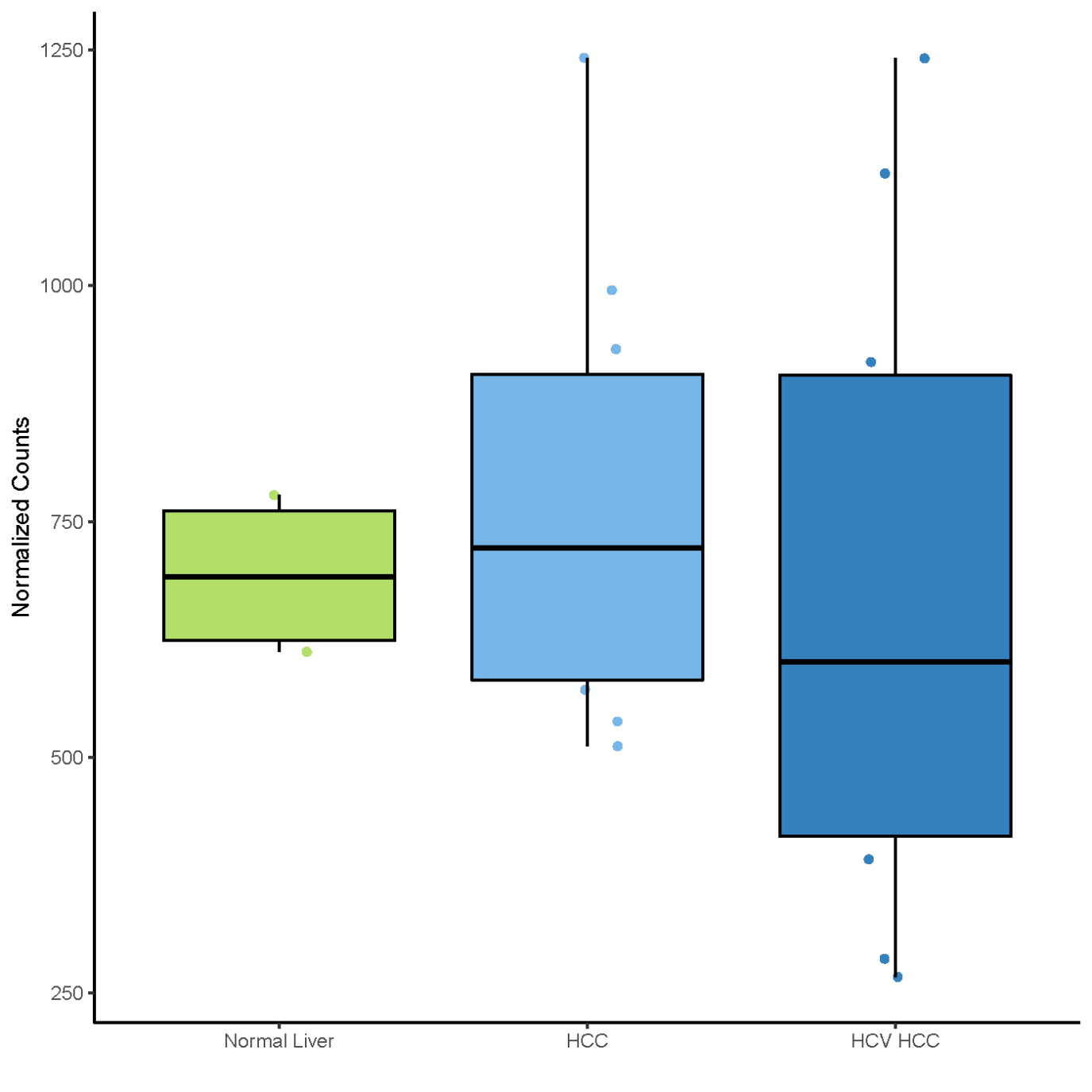


**p53**


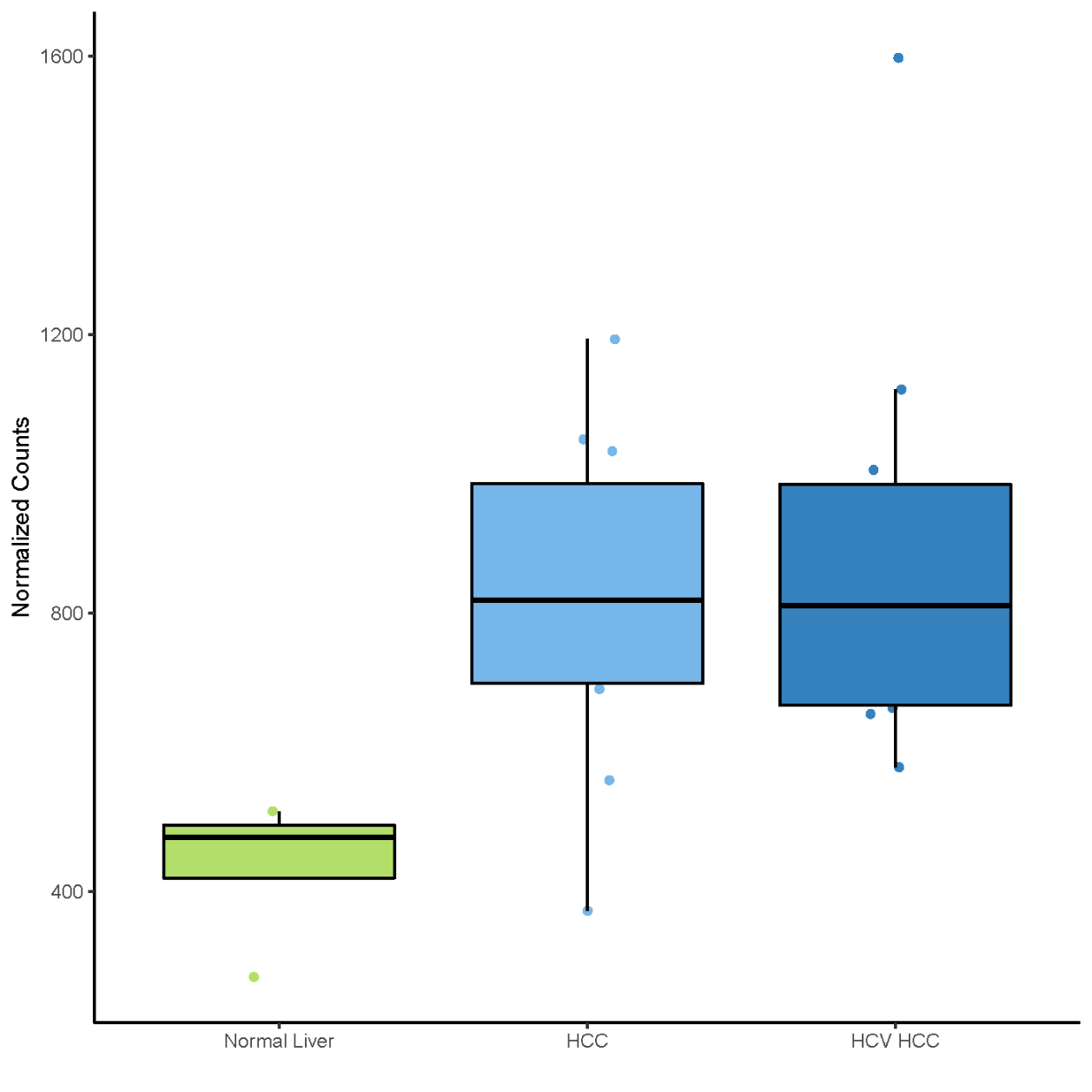

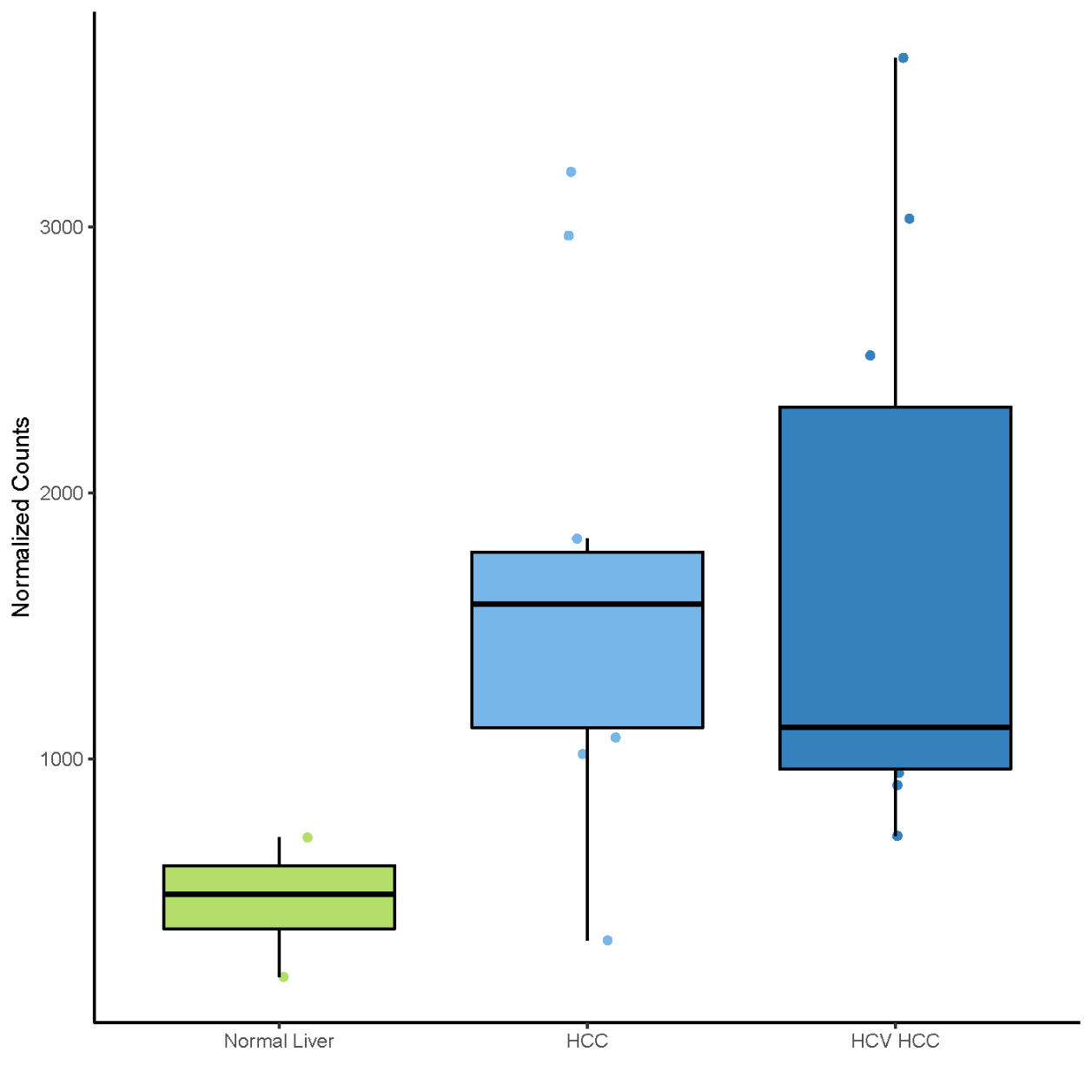


Supplementary figure 1 - continue.

**TP53BP2**

*

*

**f**

**TP53BP1**

**e**


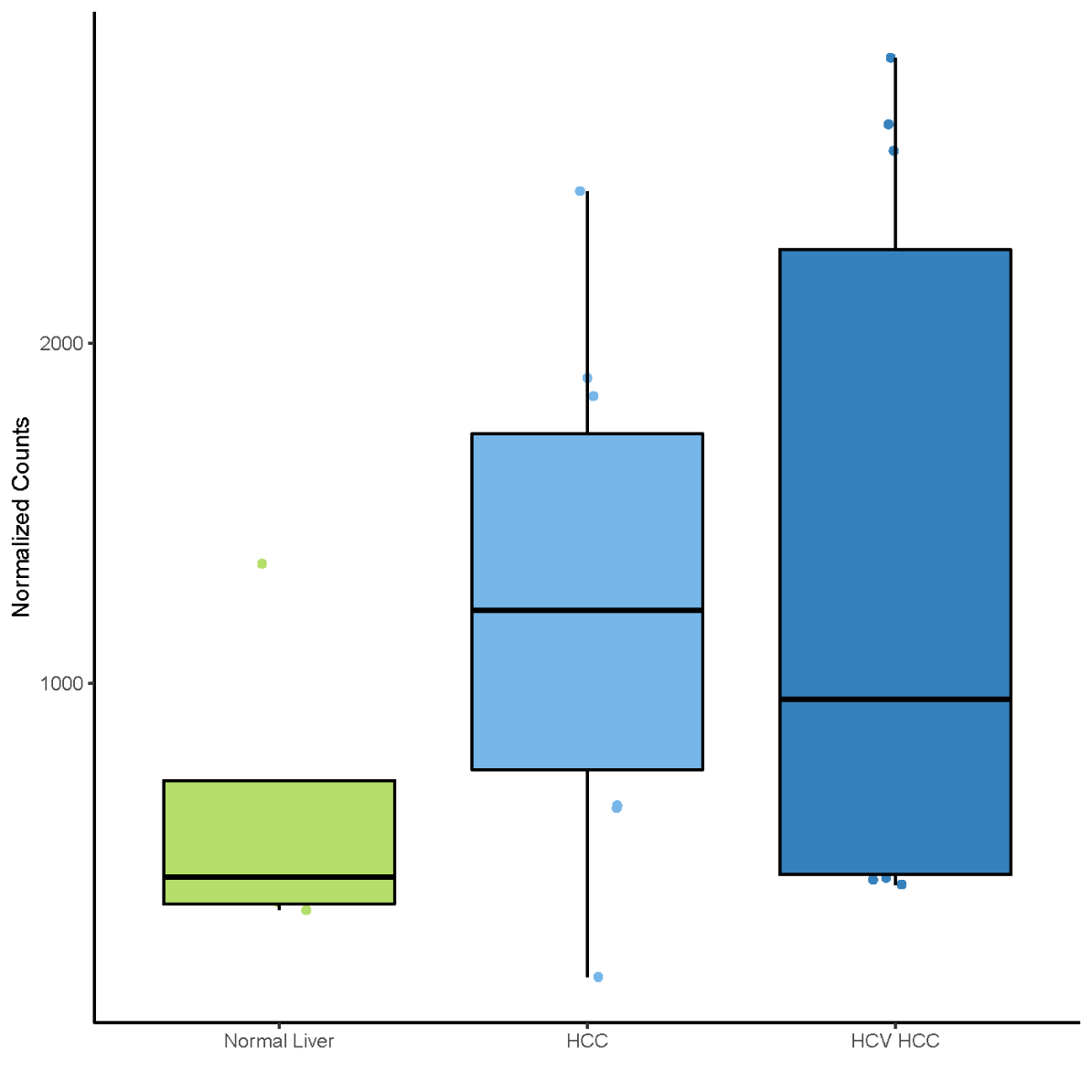

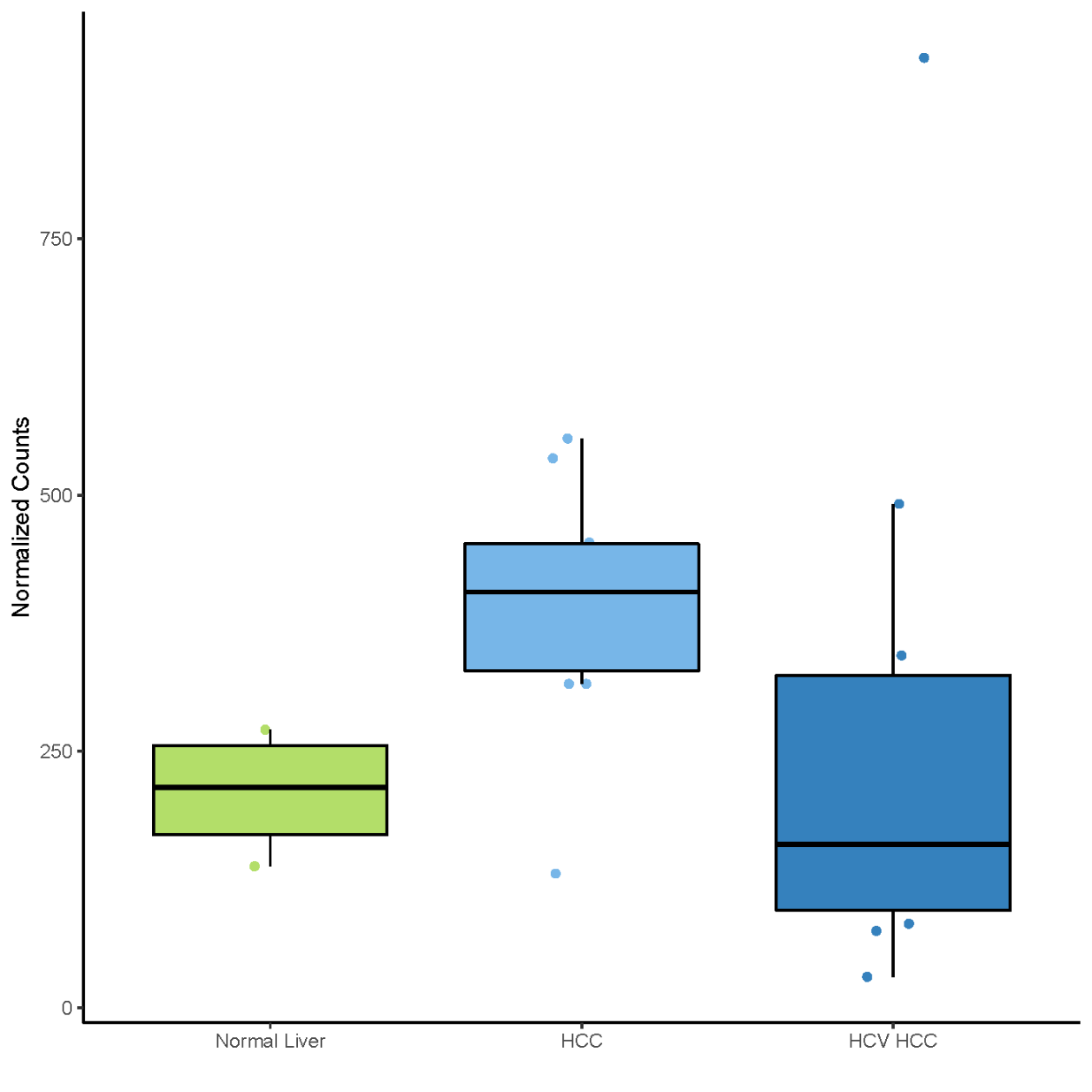


Supplementary figure 1 - continue.

**p21**

**g**

**BAX**

**h**

Supplementary figure 1 - continue.


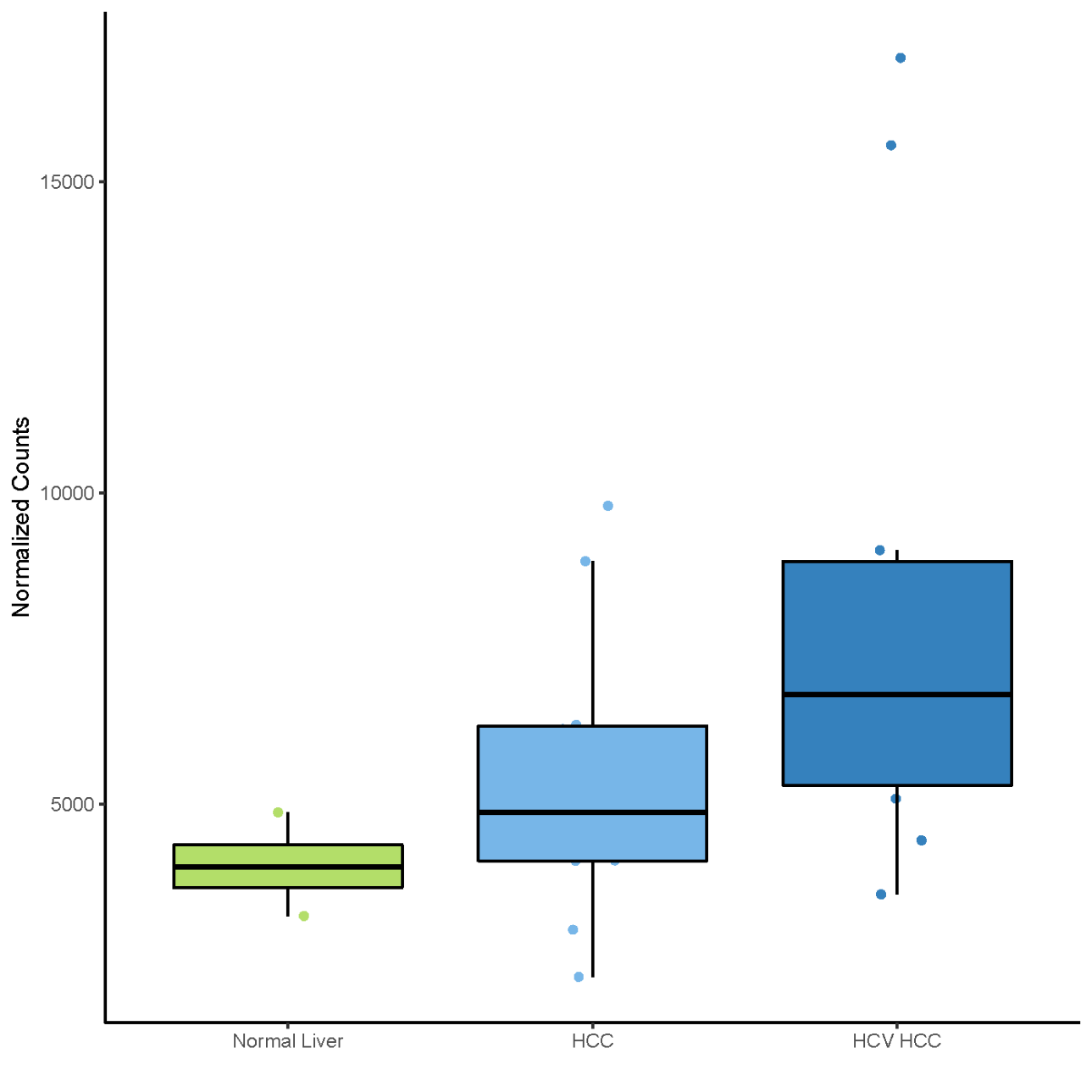


**i**

**MDM2**

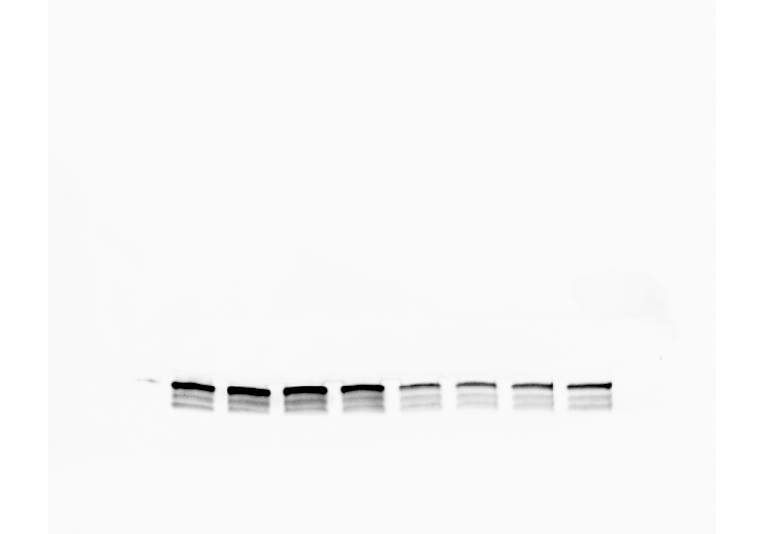

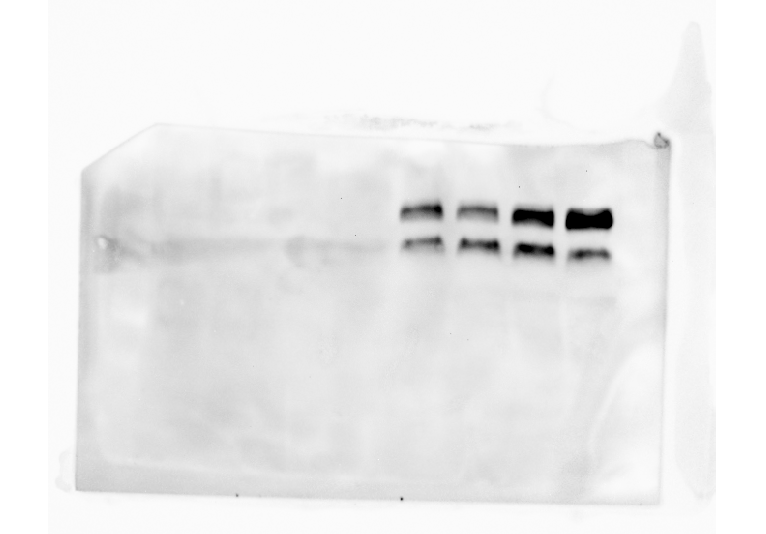


Vinculin

**a**

**b**

**c**

**d**

Supplementary figure 3.

**e**

**f**

**g**

**h**

**i**

**j**

**k**

p53

116 kDa

53 kDa

15µg

25µg

*Hep3B*

*HEK293*

*HEK293*

**Supplementary figure 4**


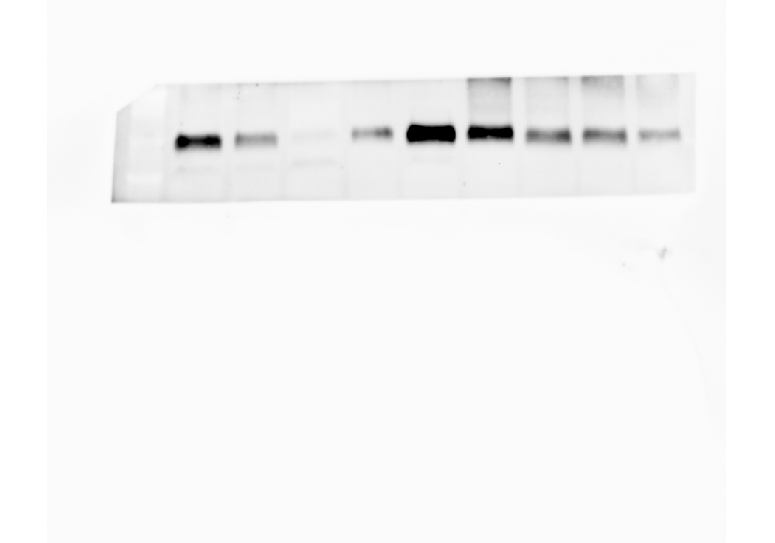

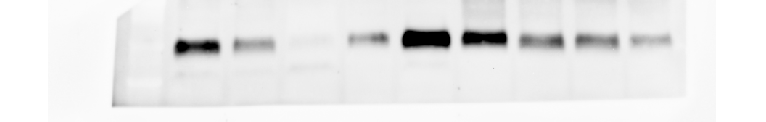


Figure 3d

Figure 2g

53 kDa

PURPL-ASO-1 + Nutlin, 3.4 µM

PURPL-ASO-1 + DOX, 300 nM

PURPL-ASO-1 + DMSO

PURPL-ASO-1

Nutlin, 3.4 µM

DOX, 300 nM

DMSO

p53-ASO

CTL-ASO

p53

Figure 2g

Figure 3d


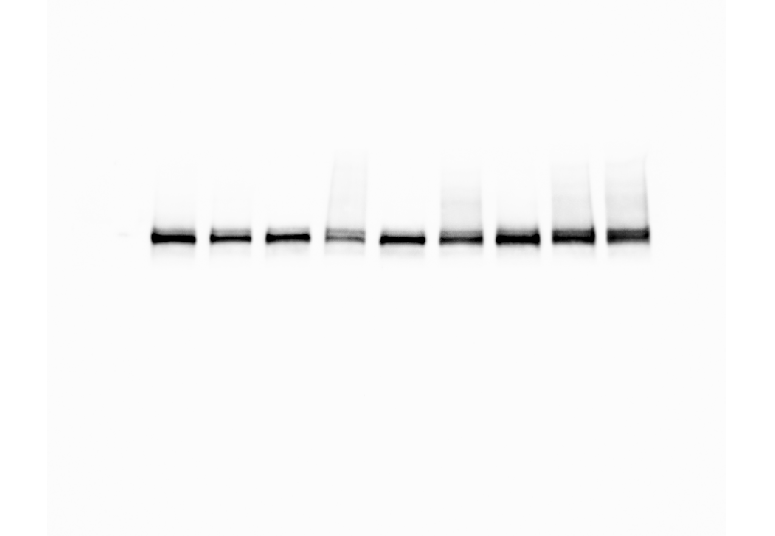

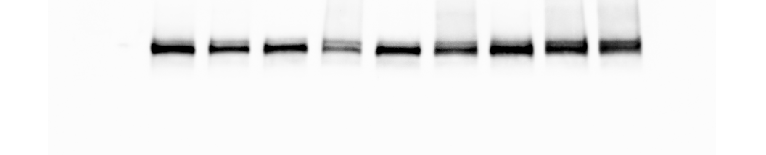


Vinculin

116 kDa

PURPL-ASO-1 + Nutlin, 3.4 µM

PURPL-ASO-1 + DOX, 300 nM

PURPL-ASO-1 + DMSO

PURPL-ASO-1

CTL-ASO

p53-ASO

DOX, 300 nM

DMSO

Nutlin, 3.4 µM

Figure 3a


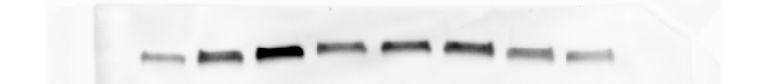


p53

DOX, 300 nM

DMSO

Nutlin, 3.4 µM

53 kDa


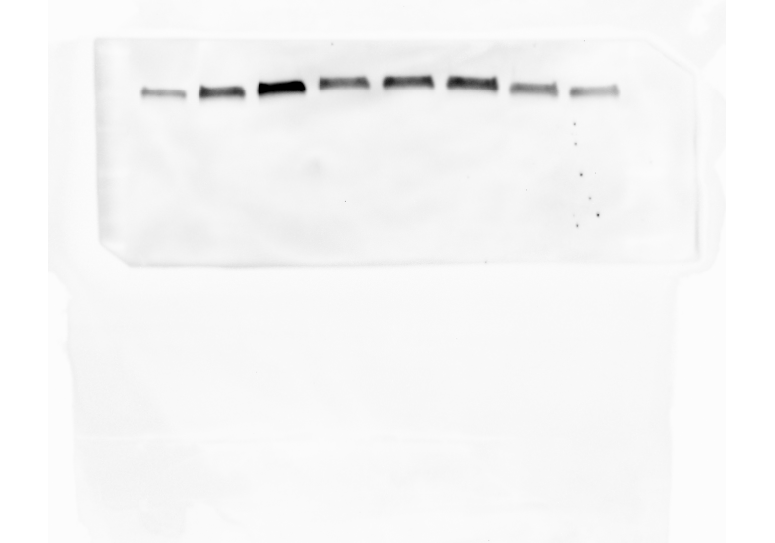


DMSO

PURPL-ASO-1+ Nutlin, 3.4 µM

PURPL-ASO-1

PURPL-ASO-1+ DOX, 300 nM

PURPL-ASO-1+ DMSO

Figure 3a


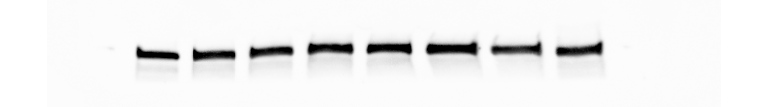


116 kDa

Vinculin

DOX, 300 nM

DMSO

DMSO

Nutlin, 3.4 µM

PURPL-ASO-1 + DMSO

PURPL-ASO-1+ DOX, 300 nM

PURPL-ASO-1+ Nutlin, 3.4 µM

PURPL-ASO-1


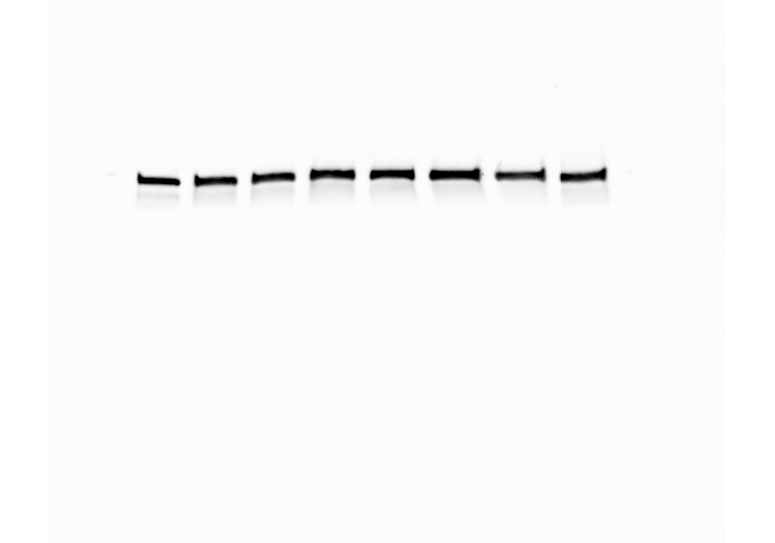


Figure 3c


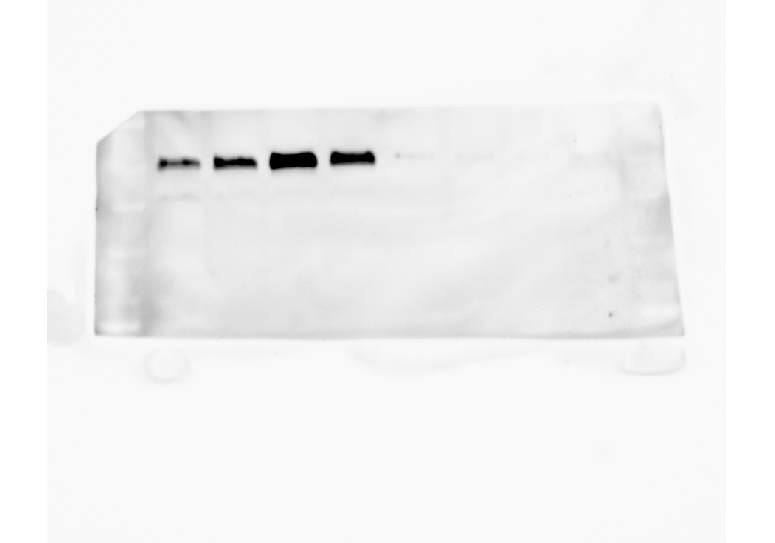

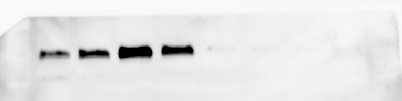


53 kDa

Nutlin, 3.4 µM

p53

DMSO

p53-ASO + DOX, 300 nM

p53-ASO + Nutlin, 3.4 µM

p53-ASO + DMSO

DOX, 300 nM

UT

DMSO

Figure 3c


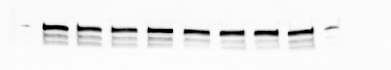


Vinculin

DMSO

UT

DOX, 300 nM

p53-ASO + DMSO

p53-ASO + Nutlin, 3.4 µM

p53-ASO + DOX, 300 nM

DMSO

116 kDa


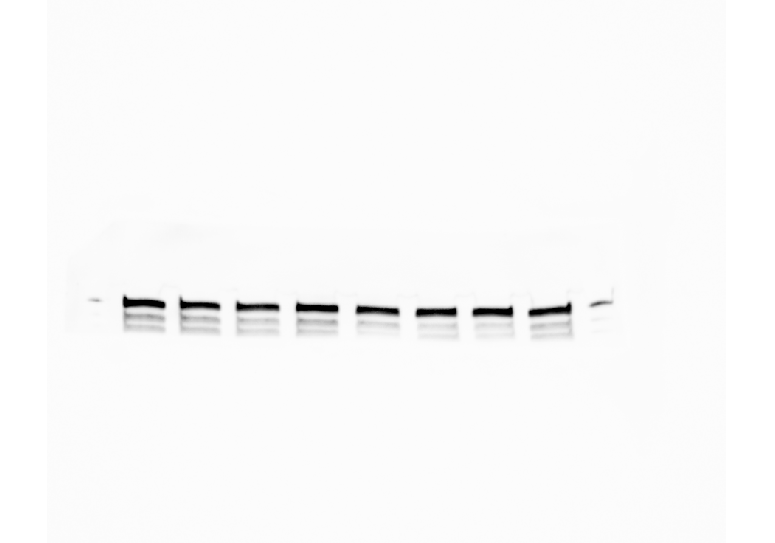


Nutlin, 3.4 µM

Figure 3f


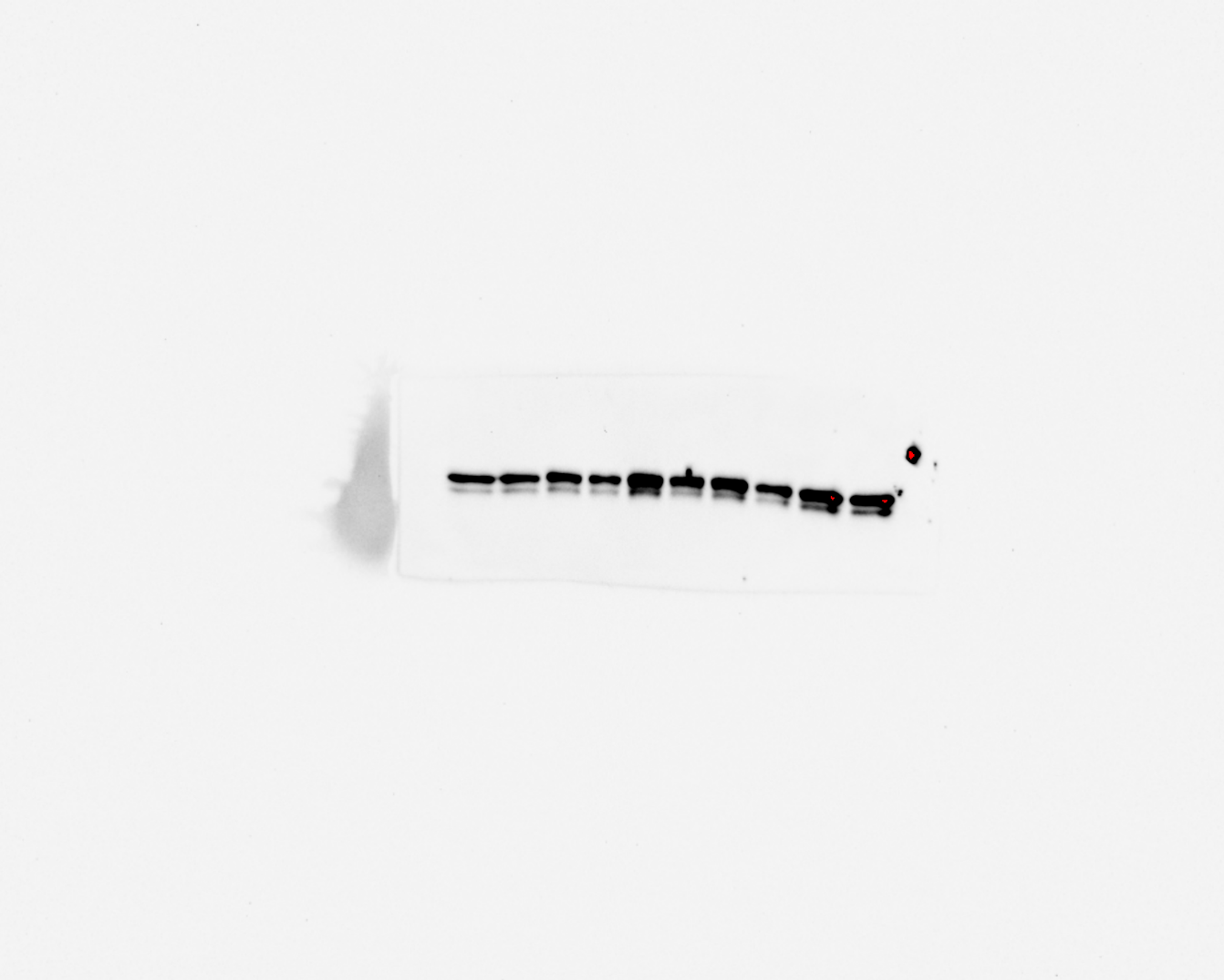


Control-ASO + DOX 3.4µM

Control-ASO + DOX 300 nM

Control-ASO + Nutlin, 3.4µM

PURPL-ASO-1 + DOX, 300 nM

PURPL-ASO-1 + Nutlin, 3.4µM

35 kDa

PURPL-ASO-1 + DMSO

UT

DMSO

DOX, 300 nM

Nutlin 3.4 µM

Caspase 3

Figure 3f


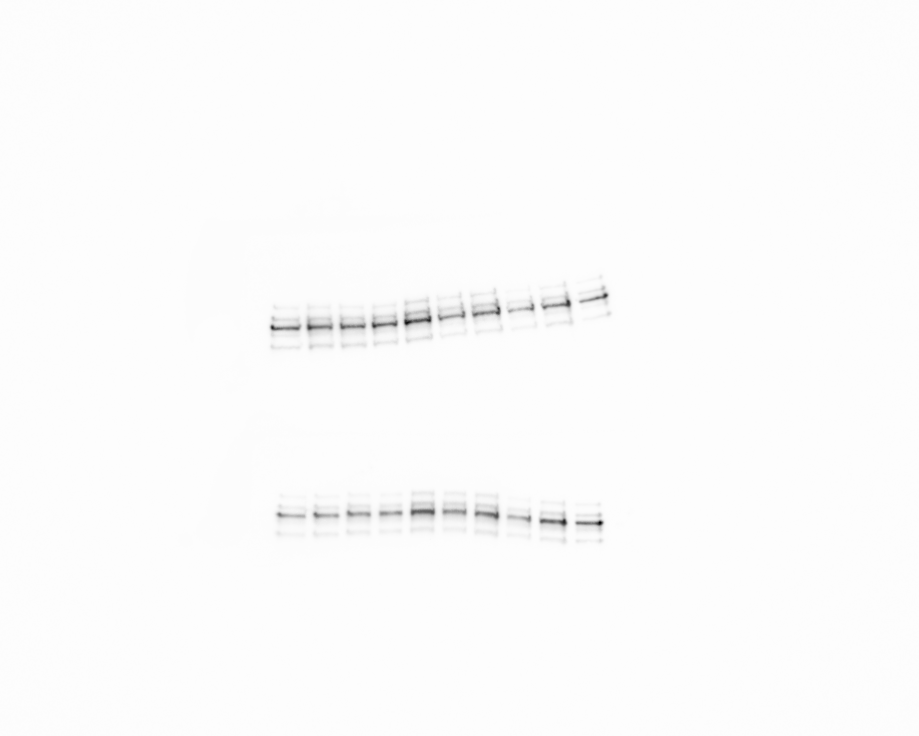


PURPL-ASO-1 + DOX, 300 nM

PURPL-ASO-1+ Nutlin, 3.4µM

Control-ASO + DOX, 300 nM

Control-ASO + Nutlin, 3.4µM

116 kDa

116 kDa

PURPL-ASO-1 + DMSO

UT

Nutlin 3.4 µM

DOX, 300 nM

DMSO

Control-ASO + DMSO

Vinculin

Upper blot from caspase 3 blot

Lower blot from caspase 7 blot

Figure 3f


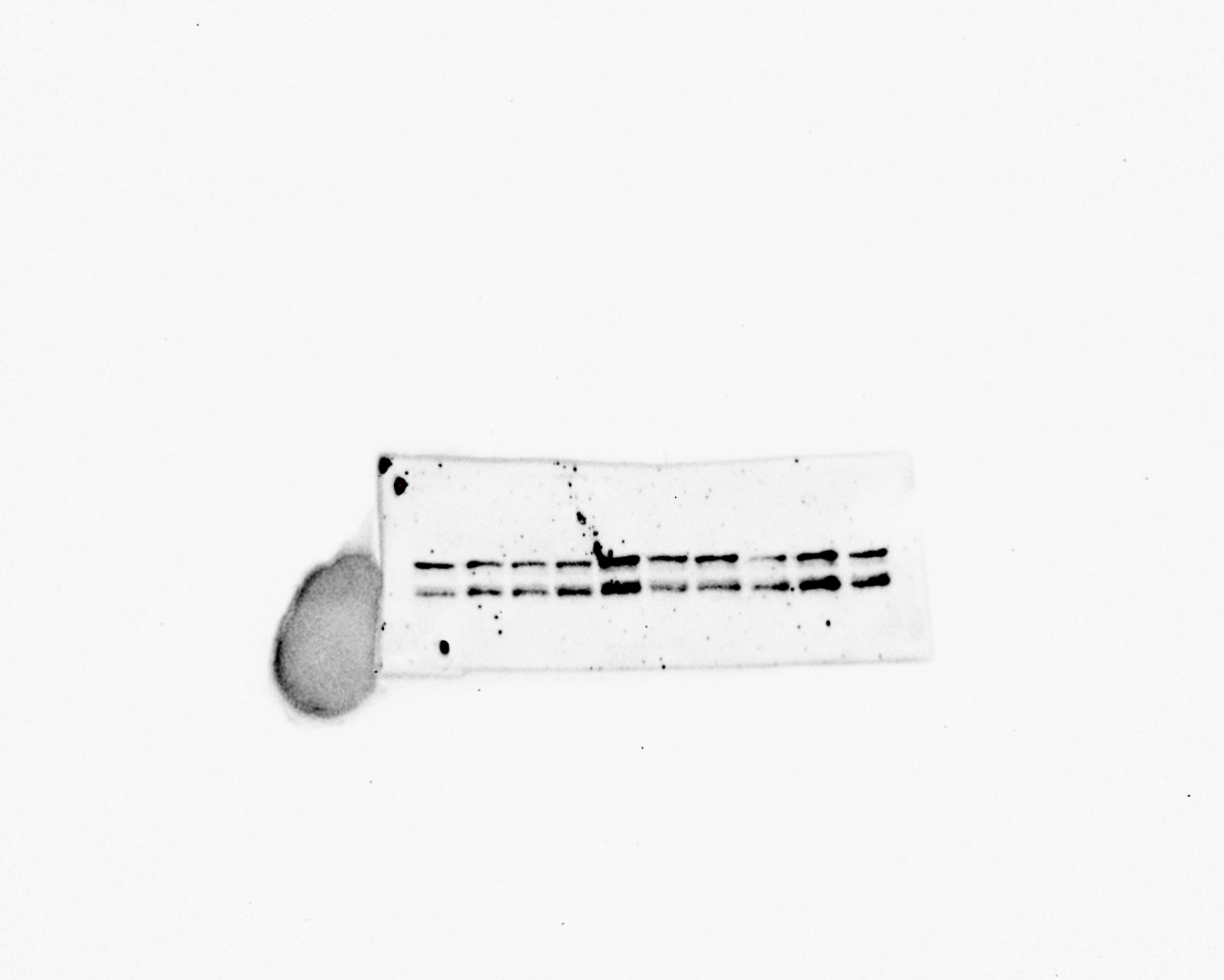


PURPL-ASO-1 + DOX, 300nM

PURPL-ASO-1 + Nutlin, 3.4µM

Control-ASO + Nutlin, 3.4µM

Control ASO + DOX, 300 nM

20 kDa

35 kDa

PURPL-ASO-1 + DMSO

Control-ASO + DMSO

Nutlin, 3.4 µM

DOX, 300 nM

DMSO

UT

Caspase 7


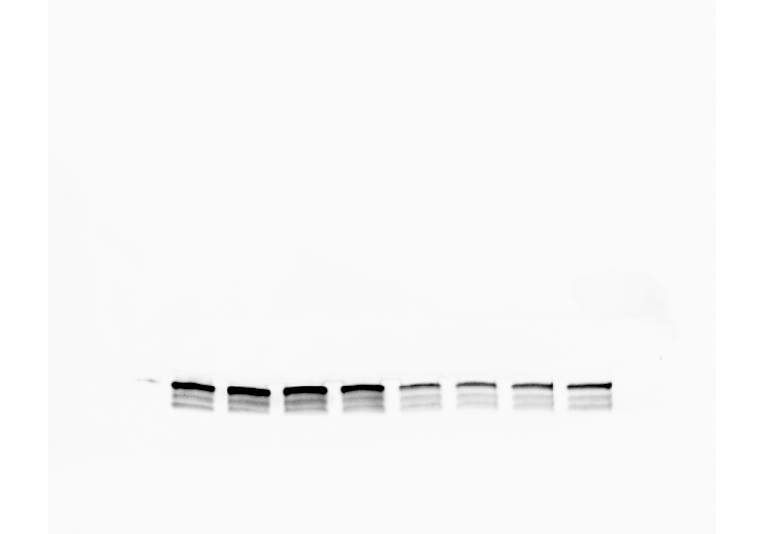


116 kDa

Sk-hep1

15µg

25µg

50µg

75µg

75µg

50µg

25µg

15µg

Hep3B

Vinculin

Supplementary Fig. S3c


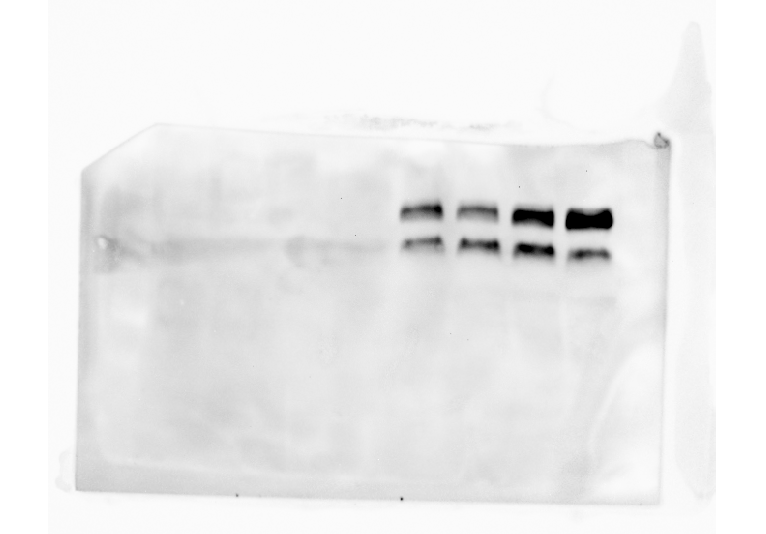


44 kDa

53 kDa

15µg

15µg

25µg

25µg

50µg

50µg

75µg

75µg

Sk-hep1

Hep3B

p53

DMSO
