# Supplementary material for: Knockdown of the long noncoding RNA PURPL induces apoptosis and sensitizes liver cancer cells to doxorubicin
Source: Sci Rep. 2022 Nov 14;12:19502. doi: 10.1038/s41598-022-23802-9 (PMC9663437; doi:10.1038/s41598-022-23802-9)
Supplement: Supplementary file 3 — Supplementary Information 3. [file 41598_2022_23802_MOESM3_ESM.pdf]

Supplementary table 2.

Assay ID	Target gene	Exon	Sequence type	Sequence
Custom design	PURPL	1-2	*Forward*	CAAGTTGAGCTTGACTGCTTAAA
			Probe	CAGGCGTTGATTGGAAATGTGTGCT
			Reverse	GGGCTTGAGAAATGAATGCAA
Hs.PT.58.39676686	TP53	1-2	*Forward*	GACACGCTTCCCTGGATTG
			Probe	AGACTGCCT TCCGGGTCACTG
			Reverse	GACGCTAGGATCTGACTGC
Hs.PT.58.39489752.g	TP53	11*	*Forward*	CCAGGACTTCCATTTGCTTTG
			Probe	TCCTCCCCACAACAAAACACCAGT
			Reverse	CTTACATCTCCCAAACATCCCT
Hs.PT.58.40874346.g	CDKN1A / p21	4-5*	*Forward*	GCAGACCAGCATGACAGAT
			Probe	TTCCTCTTGGAGAAGATCAGCCGG
			Reverse	GAGACT AAGGCAGAAGATGTAGAG
Custom design	MALAT1	1	*Forward*	GACCCTTCACCCCTCACC
			Probe	TCGATGCAGCCAGTAGCTTGGAT
			Reverse	TTATGGATCATGCCCACAAG
Hs.PT.39a.22214836	GAPDH	2-3*	*Forward*	TGT AGTTGAGGTCAATGAAGGG
			Probe	AAGGTCGGAGTCAACGGA TTTGGTC
			Reverse	ACATCGCTCAGACACCATG
Hs.PT.SSv.39858774	TBP-HEX	1-2	*Forward*	GCTGTTTAACTTCGCTTCCG
			Probe	TGATCTTTGCAGTGACCCAGCATCA
			Reverse	CAGCAACTTCCTCAATTCCTTG

** detects all variants*
