# Supplementary material for: Knockdown of the long noncoding RNA PURPL induces apoptosis and sensitizes liver cancer cells to doxorubicin
Source: Sci Rep. 2022 Nov 14;12:19502. doi: 10.1038/s41598-022-23802-9 (PMC9663437; doi:10.1038/s41598-022-23802-9)
Supplement: Supplementary file 4 — Supplementary Information 4. [file 41598_2022_23802_MOESM4_ESM.pdf]

Supplementary table 3.

Name	Target	Oligonucleotide sequence	Oligonucleotide type	Sequence length	Species	Tm (°C)
PURPL-ASO-1	PURPL	CTGatattccĉgacCG	gapmer	16	Homo sapiens	68.9
PURPL-ASO-2	PURPL	GCACaaacattagtĉgGT	gapmer	18	Homo sapiens	69.3
PURPL-ASO-3	PURPL	CGggtagtttaagtTTAG	gapmer	18	Homo sapiens	66.9
p53-ASO	TP53	CCAAatactataĉcgTC	gapmer	17	Homo sapiens	63.0
CTL-ASO	Scrambled	CGAatagttagtaGCG	gapmer	16	Homo sapiens	63.8
